# A Reverse Genetic Approach for Studying sRNAs in Chlamydia trachomatis

**DOI:** 10.1128/mbio.00864-22

**Published:** 2022-06-21

**Authors:** Kevin Wang, Lauren Sheehan, Cuper Ramirez, Asha Densi, Syed Rizvi, Roseleen Ekka, Christine Sütterlin, Ming Tan

**Affiliations:** a Department of Microbiology and Molecular Genetics, University of California, Irvine, California, USA; b Department of Developmental and Cell Biology, University of California, Irvine, California, USA; c Department of Medicine, University of California, Irvine, California, USA; National Institute of Child Health and Human Development (NICHD)

**Keywords:** MS2 affinity purification, posttranscriptional gene regulation, genetic screen, mRNA target identification, small RNA

## Abstract

sRNAs are noncoding transcripts that play critical roles in posttranscriptional regulation in prokaryotes. In the intracellular bacterium Chlamydia, sRNAs have been identified, but functional studies have been limited to an E. coli heterologous system. We have developed an inducible sRNA overexpression system in Chlamydia trachomatis and used it to screen putative sRNAs for effects on the Chlamydia developmental cycle, which involves conversion between replicating (RB) and infectious (EB) chlamydial forms. Overexpression of 4 of 13 C. trachomatis sRNAs decreased production of infectious EBs. We performed detailed characterization of CtrR3 and CtrR7, the two sRNAs that caused the largest progeny defects in our screen. By quantifying chlamydial number and infectious progeny, and by visualizing chlamydial forms using electron microscopy, we showed that overexpression of CtrR3 prevented RB-to-EB conversion, whereas CtrR7 overexpression blocked bacterial replication. We also describe a workflow that allowed us to identify the mRNA targets of CtrR3 in Chlamydia. We first used MS2 aptamer affinity purification coupled with RNA sequencing as an unbiased approach to isolate interacting mRNAs. We then prioritized candidates based on sequence complementarity to the CtrR3 target recognition sequence, which we had identified with bioinformatic and mutational analyses. Finally, we tested putative targets with translational fusion assays in E. coli and C. trachomatis. Using this integrated approach, we provide experimental evidence that YtgB and CTL0389 are mRNA targets of CtrR3 in Chlamydia. These findings demonstrate how our C. trachomatis sRNA overexpression system can be used to investigate the functions and mRNA targets of chlamydial sRNAs.

## INTRODUCTION

Chlamydia are obligate intracellular bacteria that cause a wide array of human illnesses. Chlamydia trachomatis is the most common cause of bacterial sexually transmitted disease, with more than 1.8 million new cases reported annually in the U.S. (1, 2). C. trachomatis also causes an infectious blindness called trachoma, and the related species, C. pneumoniae and C. psittaci, are responsible for community-acquired pneumonia and psittacosis, respectively (3).

All Chlamydia spp. share a developmental cycle that is marked by conversion between two specialized forms within a eukaryotic host cell (4). An infectious form, called the elementary body (EB), binds and enters the host cell. Within 2 to 8 h postinfection (hpi), the EB differentiates into a larger, intracellular form, known as the reticulate body (RB), in a membrane-bound vacuole called the chlamydial inclusion. RBs are metabolically active and undergo multiple rounds of replication before asynchronously converting back into EBs. This conversion step is critical for transmission because only EBs can infect new host cells. The time course of the intracellular infection varies between species, but for C. trachomatis, RB-to-EB conversion starts at around 24 hpi, with EBs released by 48 hpi to end the developmental cycle (5).

Another hallmark of this developmental cycle is the regulated expression of chlamydial genes in three main temporal groups (6). Early genes play important roles in establishing the inclusion, and midcycle genes are expressed during RB replication. Late genes are expressed during RB-to-EB conversion and include genes with EB-specific functions (7). For example, *hctA* and *hctB* encode histone-like proteins HctA (also known as Hc1) and HctB (or Hc2), which condense the DNA in EBs, while *omcB* encodes an EB-specific outer membrane protein (8, 9). Most of the work on chlamydial gene expression has focused on the regulation of transcription by transcription factors and alternative sigma factors (7). In contrast, little is known about the posttranscriptional regulation of gene expression in Chlamydia.

Small RNAs (sRNAs) play an important role in regulating protein expression in bacteria. These sRNAs are 50 to 500-nucleotides long and form stable secondary structures that are critical for their function. Binding of a sRNA to one or more mRNA targets through complementary base pairing commonly leads to decreased expression of each target protein (10, 11). sRNAs can be grouped into two classes (12). A *cis*-encoded sRNA is transcribed from the complementary strand of its single target gene and functions as an anti-sense RNA that binds its mRNA target to regulate its stability (13). In contrast, a *trans*-encoded sRNA typically has multiple mRNA targets and binds at or near their respective ribosome binding site (RBS). This association occurs via a short region of imperfect sequence complementarity that impedes ribosome binding and/or promotes mRNA degradation. Target genes of *trans*-encoded sRNAs are located at different genomic sites from the sRNA, which makes their identification more challenging (12).

Putative chlamydial sRNAs have been identified, but functional analysis has been limited to a heterologous system. Albrecht et al. and AbdelRahman et al. used RNA sequencing or an intergenic tiling microarray to identify a total of 66 putative sRNAs in C. trachomatis, 20 of which were confirmed by Northern blotting (3 *cis* and 17 *trans* sRNAs) (14, 15). Functional studies have been performed on two C. trachomatis sRNAs, IhtA and CTIG270. IhtA is a *trans*-encoded sRNA that decreased protein, but not transcript levels of its target HctA when both were co-expressed in E. coli (16). CTIG270, in contrast, is a *cis*-encoded sRNA that downregulated transcript and protein expression of the peptidoglycan synthesis gene *ftsI* in an E. coli co-expression assay (14, 17). The functions and targets of these sRNAs have not been assessed in Chlamydia with its complex biphasic developmental cycle.

In this study, we describe a novel C. trachomatis sRNA overexpression system to study chlamydial sRNAs in their native environment. We used this genetic approach to screen 13 chlamydial sRNAs for deleterious effects on the infection and identified 4 whose overexpression caused a severe reduction in infectious progeny. We also applied our genetic system to identify mRNA targets of a chlamydial sRNA. By combining an unbiased screen to capture putative mRNA targets with a bioinformatics-based prioritization scheme and functional studies in E. coli and Chlamydia, we identified YtgB and CTL0389 as likely mRNA targets of the uncharacterized sRNA CtrR3.

## RESULTS

### Development of a sRNA overexpression system in Chlamydia.

**FIG 1:**
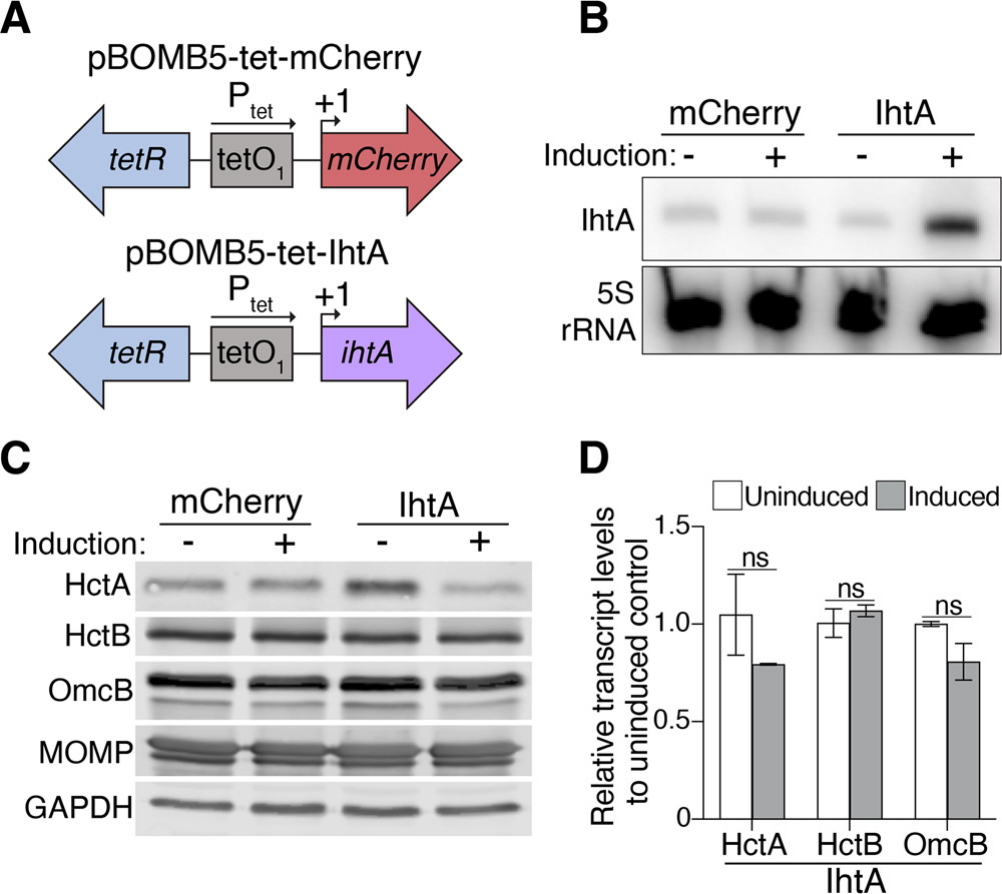
Development of an inducible sRNA overexpression system in C. trachomatis. (A) Schematics of the overexpression cassettes for mCherry (control) and IhtA in the pBOMB5 plasmid. +1 marks the transcriptional start site. (B) Northern blot of HeLa cells infected with Chlamydia transformants that overexpressed either mCherry or IhtA after incubation with or without 50 ng/mL aTc starting at 16 hpi. IhtA levels are shown at 36 hpi. 5S rRNA served as a loading control. (C) HeLa cells infected for 36 h with mCherry or IhtA transformants that were either left uninduced or induced with aTc at 16 hpi were lysed and subjected to Western blotting with antibodies to HctA, HctB, and OmcB. MOMP and GAPDH served as loading controls for Chlamydia and host cells, respectively. (D) HeLa cells infected with the IhtA transformant and induced as above were analyzed by RT-qPCR for the transcript levels of HctA, HctB, and OmcB at 36 hpi. 16S rRNA served as the reference gene. Transcript levels for each gene in the induced sample was compared to the average transcript levels of each respective gene in the uninduced control via the Pfaffl equation. Data are presented as mean ± SEM (*n* = 3).

To study the role of chlamydial sRNAs in the developmental cycle, we developed an inducible system to overexpress individual sRNAs in C. trachomatis. This system is based on the pBOMB4 plasmid, a chlamydial shuttle vector for tetracycline-inducible protein expression in C. trachomatis (18). We modified pBOMB4 by removing the second downstream Tet operator to avoid adding extra nucleotides to the 5′ end of the sRNA, which might alter its secondary structure and function (Fig. 1A). The sRNA overexpression cassette was also relocated to avoid possible read-through transcription of downstream genes. We called this new plasmid pBOMB5 (Fig. S1).

10.1128/mbio.00864-22.1 FIG S1 Plasmid map of pBOMB5-tet-sRNA. The sRNA expression cassette was moved to prevent potential transcriptional read-through into the *bla* gene. The IncG terminator was placed downstream of the *bla* gene to prevent read-through into the sRNA expression cassette. Download FIG S1, PDF file, 0.4 MB. Copyright © 2022 Wang et al. 2022 Wang et al. https://creativecommons.org/licenses/by/4.0/ This content is distributed under the terms of the Creative Commons Attribution 4.0 International license.

We tested this overexpression system with IhtA, the best characterized chlamydial sRNA, which was previously shown to downregulate translation of the histone-like protein HctA in a heterologous system (16). We cloned the sequence of IhtA into this plasmid to generate pBOMB5-tet-IhtA and transformed it into C. trachomatis (Fig. 1A). Induction with anhydrotetracycline (aTc) increased IhtA expression, as measured by Northern blots (Fig. 1B) and decreased protein levels of HctA, but not HctB and OmcB, which are two other late gene products (Fig. 1C). These effects were specific for IhtA because they were not seen when mCherry expression was induced by aTc in the control transformant (data not shown). IhtA overexpression did not change mRNA levels of HctA, HctB, or OmcB (Fig. 1D), demonstrating that IhtA negatively regulates the protein, but not transcript levels of HctA in Chlamydia. These results provide proof of principle that our overexpression system offers a novel and powerful approach to alter the levels of a sRNA and its target inside this intracellular bacterium.

### Identification of sRNAs that are involved in a productive chlamydial infection.

**FIG 2:**
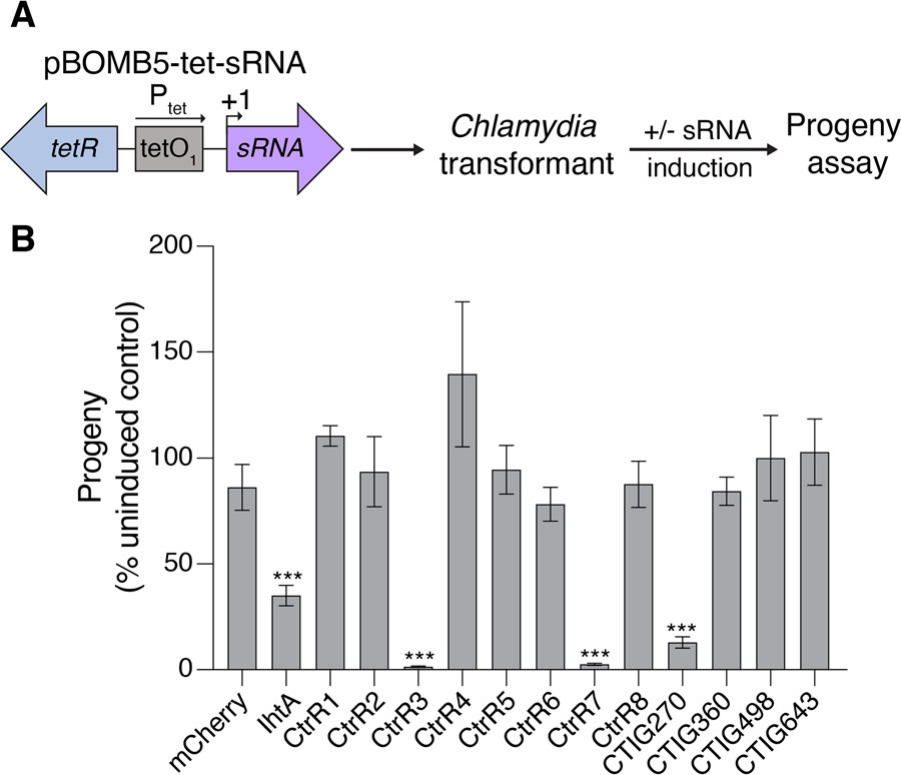
Screen to identify C. trachomatis sRNAs important for the Chlamydia developmental cycle. (A) Schematic of the sRNA overexpression screen. (B) For each transformant, the number of infectious EBs of induced samples is expressed as a percentage of the uninduced control samples. HeLa cells infected with transformants expressing mCherry or a sRNA were incubated in the absence or presence of 50 ng/mL aTc starting at 1 hpi. Lysates were collected at 32 hpi and analyzed for infectious progeny production in a secondary infection. Data are presented as mean ± SEM (*n* = 3), ***, *P < *0.001.

We next used this sRNA overexpression system to screen putative C. trachomatis sRNAs for effects on the developmental cycle. We focused on 14 sRNAs that have been previously confirmed by Northern blotting analysis and whose transcription start sites have been mapped (14, 15). Each sRNA was cloned into pBOMB5, and 12 of 14 were successfully transformed into C. trachomatis. We then individually induced the overexpression of these 12 sRNAs, as well as IhtA, and measured effects on the production of infectious EBs with a progeny assay, which is performed with a secondary infection (Fig. 2A).

Overexpression of four sRNAs caused measurable reductions in progeny (Fig. 2B). The largest defect was with CtrR3 overexpression (Fig. S2A), which decreased progeny by 68-fold at 32 hpi, compared to its uninduced control. The severity of the CtrR3 defect was proportional to the aTc concentration used for induction (Fig. S3). There were also overexpression defects with CtrR7 (Fig. S2B), which decreased progeny by 38-fold at 32 hpi, and with IhtA and CTIG270, which caused more modest 2.8-fold and 7.7-fold reductions in progeny, respectively (Fig. 2B). Induction of the nine other sRNAs did not affect progeny, and of these, we verified overexpression for CtrR1 and CtrR4 by Northern blotting analysis (Fig. S2C). These results suggest that CtrR3, CtrR7, CTIG270 and IhtA have important functions in the chlamydial developmental cycle.

10.1128/mbio.00864-22.2 FIG S2 Overexpression of CtrR3, CtrR7, CtrR1, and CtrR4. Northern blots of HeLa cells infected with (A) CtrR3, (B) CtrR7, and (C) CtrR1 or CtrR4 transformants. CtrR3, CtrR1, and CtrR4 transformants were induced at 1 hpi and analyzed at the indicated time points. CtrR7 transformant was induced at 16 hpi and analyzed at 32 hpi because CtrR7 overexpression from 1 hpi strongly impacted bacterial growth. CtrR3, CtrR7, CtrR1 and CtrR4 levels are shown; and 5S rRNA served as a loading control. Download FIG S2, PDF file, 0.5 MB. Copyright © 2022 Wang et al. 2022 Wang et al. https://creativecommons.org/licenses/by/4.0/ This content is distributed under the terms of the Creative Commons Attribution 4.0 International license.

10.1128/mbio.00864-22.3 FIG S3 CtrR3 overexpression affects progeny in a dose-dependent manner. HeLa cells were infected with CtrR3 transformants and incubated with different amount of aTc at 1 hpi. aTc was replenished at 16 hpi, and samples were analyzed at 32 hpi. The number of infectious EBs in the induced conditions is expressed as a percentage of the number of EBs in uninduced control samples. Download FIG S3, PDF file, 0.4 MB. Copyright © 2022 Wang et al. 2022 Wang et al. https://creativecommons.org/licenses/by/4.0/ This content is distributed under the terms of the Creative Commons Attribution 4.0 International license.

### sRNA overexpression can disrupt different steps in the developmental cycle.

**FIG 3:**
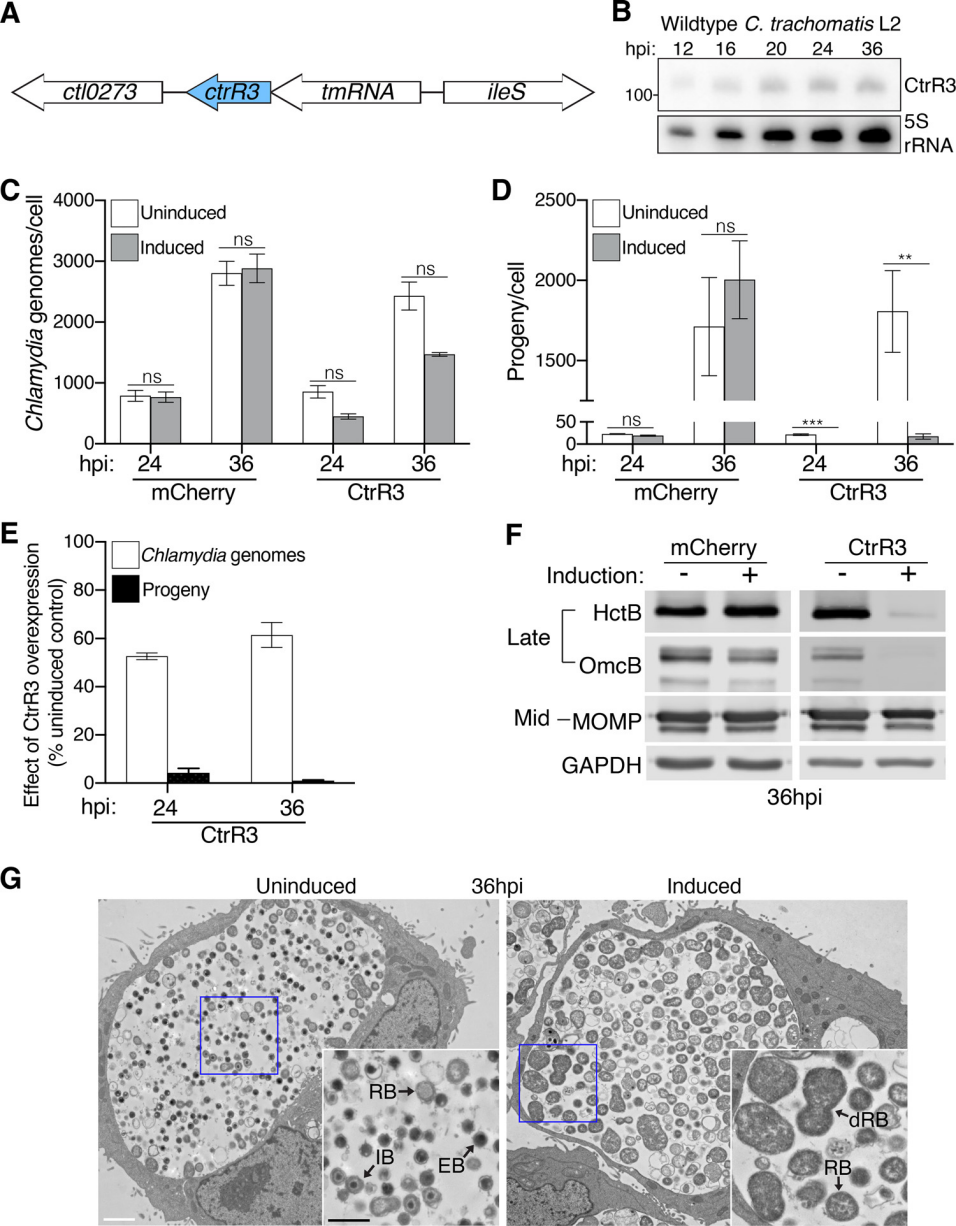
CtrR3 overexpression results in an RB-to-EB conversion defect. (A) The position of the *ctrR3* gene in the C. trachomatis serovar L2 genome is shown. (B) Total RNA isolated from HeLa cells that were infected with wild type C. trachomatis serovar L2 was analyzed by Northern blotting at the indicated times. Blots were probed for CtrR3 and 5S rRNA as loading control. (C) The number of chlamydial genomes and (D) infectious EBs produced in HeLa cells infected either with the mCherry or the CtrR3 transformant were determined by qPCR and progeny assay, respectively, at the indicated time points and normalized to the number of host cells. mCherry or CtrR3 overexpression was induced as described in Fig. 2B. Data are mean ± SEM (*n* = 3); **, *P ≤ *0.01 and ***, *P < *0.001. (E) The numbers of chlamydial genomes and infectious EBs in CtrR3 overexpression samples are expressed as a percentage of their respective uninduced control to represent the relative effect of CtrR3 overexpression. (F) Western blot analysis of lysates from HeLa cells infected with the mCherry or CtrR3 transformants treated with aTc from 1–36 hpi. The levels of HctB and OmcB (late gene products) MOMP (mid gene product) are shown. GAPDH served as a loading control. (G) Electron micrographs of CtrR3 transformant-infected HeLa cells at 36 hpi, in the absence or presence of aTc starting at 1 hpi. The chlamydial developmental forms are as indicated: RB, reticulate body; dRB, dividing reticulate body; IB, intermediate body; and EB, elementary body. White scale bar: 2 μm, black scale bar: 1 μm.

**(i) CtrR3 overexpression caused an RB-to-EB conversion defect.** We performed additional experiments to identify the stage in the developmental cycle disrupted by CtrR3 overexpression. This *trans-*encoded sRNA is located downstream of the tmRNA, and we detected its endogenous expression throughout the developmental cycle (Fig. 3A and B; Fig. S4A). RT-qPCR was not used to quantify CtrR3 expression because the sRNA is only ~110 nt long. Compared to its uninduced control, CtrR3 overexpression only had a modest effect on RB replication, as shown by 1.9-fold and 1.6-fold decreases in genome copy number at 24 hpi and 36 hpi, respectively (Fig. 3C). In contrast, there was a 24-fold and 104-fold reduction in progeny at 24 and 36 hpi, respectively (Fig. 3D), and thus a disproportionate effect on EB production compared to RB replication (Fig. 3E). Progeny counts remained lower also at later times, which indicates that this defect was not due to a delay in EB production (48 and 60 hpi, data not shown).

10.1128/mbio.00864-22.4 FIG S4 Quantification of endogenous CtrR3 and CtrR7 expression. (A) Top: Total RNA extracted from HeLa cells infected with wild type C. trachomatis serovar L2 was analyzed by northern blotting at the indicated times. Bottom: Quantification of northern blot in (A). CtrR3 was normalized to 5S at each time point. (B) HeLa cells infected with wild type C. trachomatis serovar L2 were analyzed by either RT-qPCR for the transcript levels of CtrR7 or qPCR for chlamydial genome number at the indicated time points. For each time point, CtrR7 transcript levels were first compared to the CtrR7 level of the 1 hpi sample via the Pfaffl equation and subsequently normalized to the chlamydial genome number. Data are shown as mean ± SEM (*n* = 2). Download FIG S4, PDF file, 0.4 MB. Copyright © 2022 Wang et al. 2022 Wang et al. https://creativecommons.org/licenses/by/4.0/ This content is distributed under the terms of the Creative Commons Attribution 4.0 International license.

We confirmed the defect in EB production with additional assays. Western blot analysis at 36 hpi showed that CtrR3 overexpression decreased expression of the EB-specific proteins HctB and OmcB, but not of MOMP, which is present in RBs and EBs (Fig. 3F). Transmission electron microscopy analysis of these samples at 36 hpi revealed inclusions that were full of RBs and dividing RBs, but only had few EBs and intermediate bodies (IBs), which are RBs in the process of converting into EBs (Fig. 3G). This distribution of chlamydial developmental forms was strikingly different from uninduced control cells, which resembled a wild-type infection in having predominantly EBs at this late time (5). Together, these findings provide strong evidence that the large progeny defect caused by CtrR3 overexpression is due to impairing RB-to-EB conversion.

**FIG 4:**
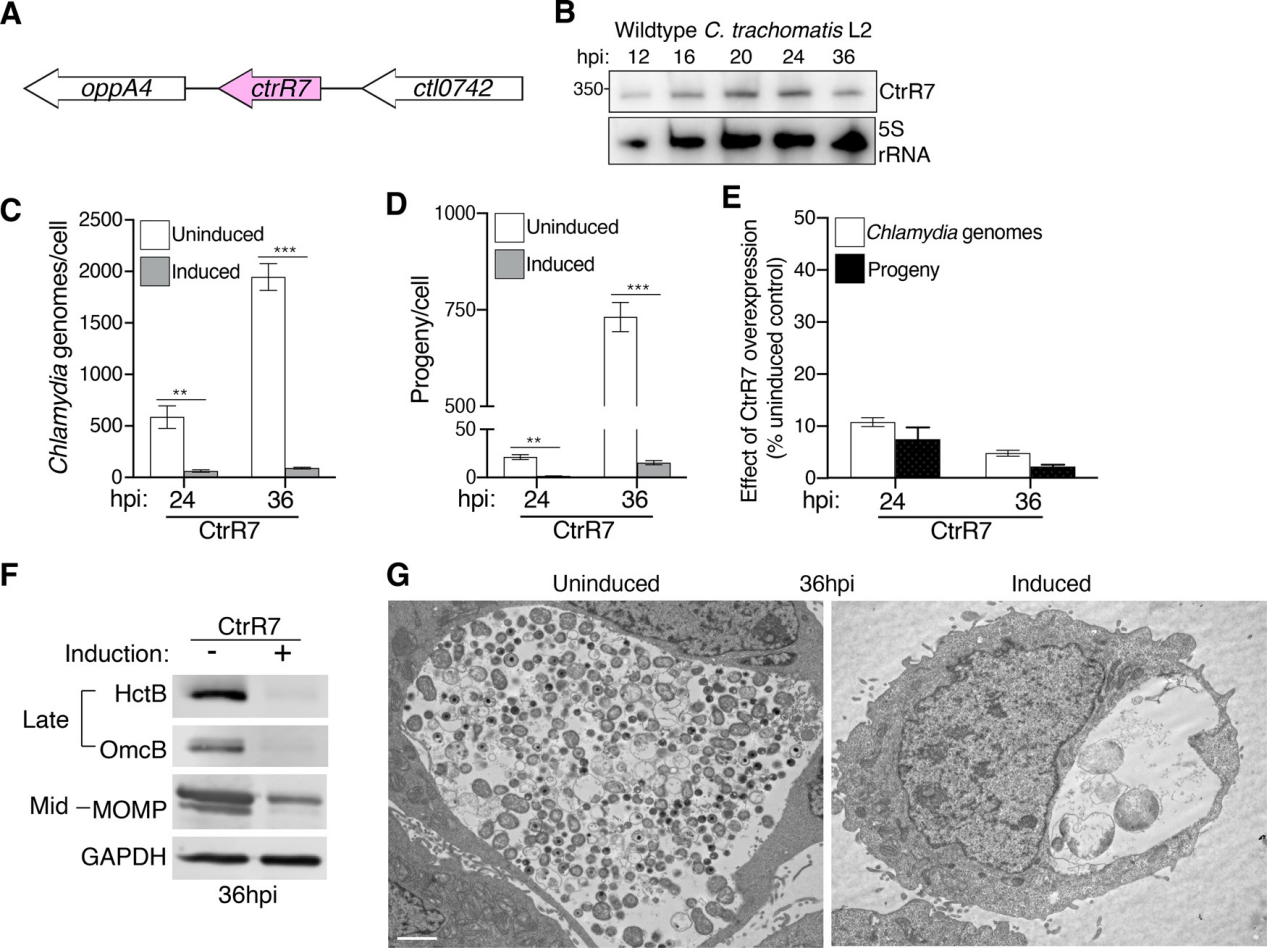
CtrR7 overexpression results in RB replication defect. (A) The position of the *ctrR7* gene in the C. trachomatis serovar L2 genome is shown. (B) Total RNA extracted from HeLa cells that were infected with wild type C. trachomatis serovar L2 was analyzed by Northern blotting at the indicated times. Blots were probed for CtrR7 and 5S rRNA as loading control. (C) The number of chlamydial genomes and (D) infectious EBs produced in HeLa cells infected with the CtrR7 transformant were determined by qPCR and progeny assay, respectively, at the indicated time points and normalized to the number of host cells. CtrR7 overexpression was induced as described in Fig. 2B. Data are mean ± SEM (*n* = 3); **, *P ≤ *0.01 and ***, *P < *0.001. (E) The numbers of chlamydial genomes and infectious EBs in CtrR7 overexpression samples are expressed as a percentage of their respective uninduced control to represent the relative effect of CtrR7 overexpression. (F) Western blot analysis of lysates from HeLa cells infected with the CtrR7 transformants treated with aTc from 1 to 36 hpi. The levels of HctB and OmcB (late gene products) MOMP (mid gene product) are shown. GAPDH served as a loading control. (G) Electron micrographs of CtrR7 transformant-infected HeLa cells at 36 hpi, in the absence or presence of aTc starting at 1 hpi. White scale bar: 2 μm.

**(ii) CtrR7 overexpression caused an RB replication defect.** CtrR7 is a *trans*-encoded sRNA that is located in the intergenic region between the *oppA4* and *ctl0742* genes (Fig. 4A). Its endogenous expression follows a mid-cycle pattern, with a peak at 16 hpi and a decrease at 36 hpi (Fig. 4B; Fig. S4B). In contrast to CtrR3, CtrR7 overexpression had strong effects on both RB replication (9.3-fold and 21-fold decreases in chlamydial genomes at 24 and 36 hpi, respectively, Fig. 4C) and infectious EB production (14-fold and 47-fold decreases in progeny at 24 and 36 hpi, respectively, Fig. 4D). These defects in chlamydial replication and EB production were proportional to each other (Fig. 4E). Western blot analysis further demonstrated that CtrR7 overexpression affected the expression of both late (HctB and OmcB) and midcycle gene products (MOMP) (Fig. 4F). In addition, transmission electron microscopy at 36 hpi showed that CtrR7 overexpression resulted in an inclusion that was mostly empty except for a few large RBs that resemble aberrant bodies observed under stress-induced persistence (Fig. 4G). Thus, the severe reduction in progeny caused by CtrR7 overexpression can be mainly ascribed to an RB replication defect.

Together, these analyses of CtrR3 and CtrR7 demonstrate that overexpression of a chlamydial sRNA can cause a progeny defect by disrupting different steps in the developmental cycle.

### Identification of the target recognition sequence of CtrR3.

**FIG 5:**
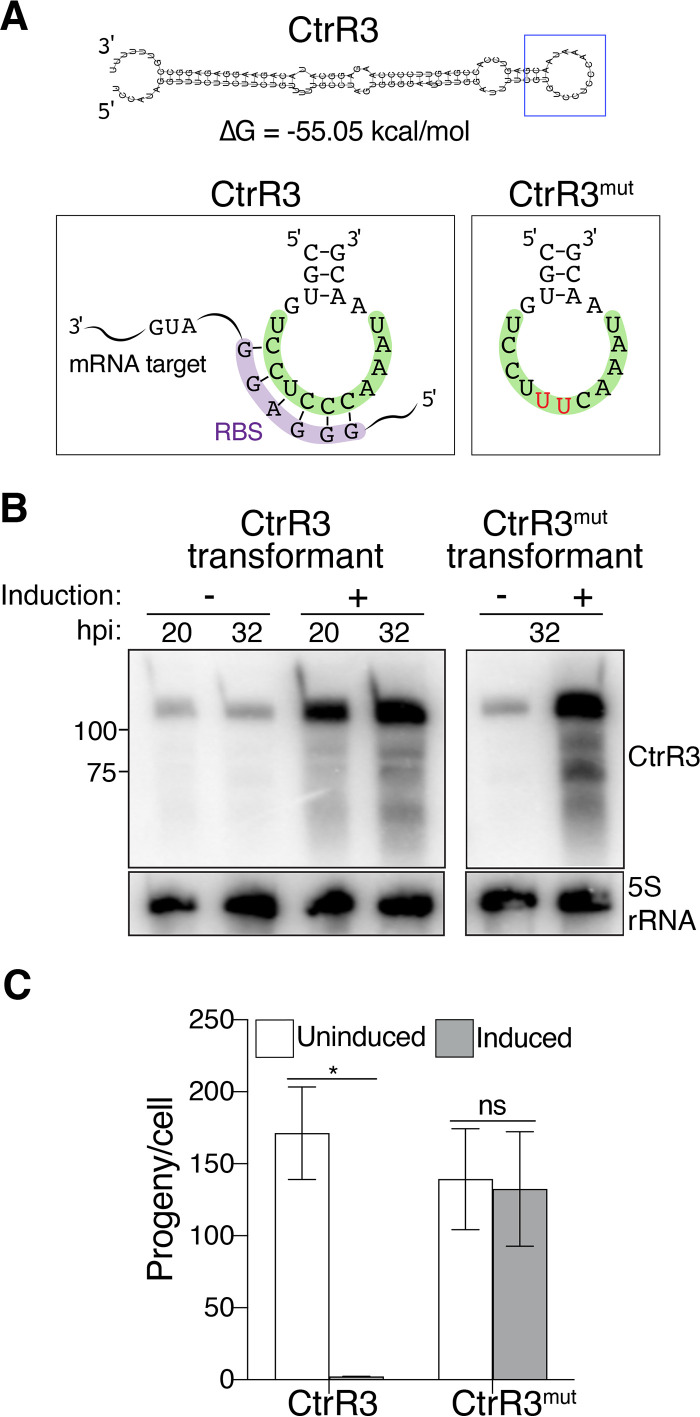
The C-rich hairpin sequence is necessary for CtrR3 function. (A) The secondary structure of CtrR3 was predicted bioinformatically using the RNAfold software. The free energy of the thermodynamic ensemble is shown (ΔG). The box on the left shows its C-rich hairpin sequence (highlighted in green) interacting with the RBS (highlighted in purple) of a putative mRNA target. The box on the right box shows the sequence for CtrR3^mut^ with two C-to-U substitutions. (B) Total RNA extracted at the indicated time points from the CtrR3 or the CtrR3^mut^ transformant in the absence or presence of aTc was analyzed by Northern blotting. CtrR3 and CtrR3^mut^ levels were detected by a probe that recognized both forms of the sRNA; 5S rRNA served as the loading control. (C) The numbers of infectious EBs in HeLa cells infected with either CtrR3 or CtrR3^mut^ transformants after treatment with aTc from 1 hpi were determined by progeny assay at 32 hpi and normalized to the number of host cells. Data are shown as mean ± SEM (*n* = 3); *, *P ≤ *0.05.

We next applied our overexpression system to identify mRNA targets of a chlamydial sRNA, exemplified by CtrR3, which is the sRNA that caused the greatest progeny defect. We first used a combined bioinformatic and mutational approach to identify the target recognition sequence, or seed region, of CtrR3. From the predicted secondary structure (RNAfold) (19) of CtrR3, we predicted the seed region to be in the large C-rich single-stranded hairpin loop because of its sequence complementarity to the ribosome binding site (RBS) sequence of bacterial mRNAs (Fig. 5A). In addition, the sequence is conserved in CtrR3 from *C. suis* and C. muridarum (data not shown). To test if this sequence is important for CtrR3 function, we generated a transformant expressing CtrR3 with two C-to-U substitutions within the potential anti-RBS sequence (CtrR3^mut^) (Fig. 5A). The two nucleotide substitutions do not alter the predicted secondary structure of the sRNA (data not shown). Overexpression of CtrR3^mut^ (Fig. 5B) no longer decreased progeny production (Fig. 5C), providing strong experimental evidence that this hairpin loop sequence is the seed region through which CtrR3 mediates its effects on EB production.

### Identification of mRNA targets of CtrR3.

For the remainder of this study, we used CtrR3 to develop a generalizable, multistep approach to isolate and identify the mRNA targets of a sRNA in Chlamydia.

**(i) MS2-affinity purification with RNA sequencing (MAPS).** To identify mRNAs that bind to CtrR3, we overexpressed CtrR3 tagged at the 5′ end with two MS2 hairpins (MS2-CtrR3). We used RNAfold (19) to check that these MS2 hairpins did not alter the predicted secondary structure of CtrR3 (Fig. S5A). We also constructed a control consisting of two MS2 hairpins followed by a rnpB T1 terminator (MS2-Control, Fig. S5A). MS2-CtrR3 and MS2-Control were individually cloned into pBOMB5 and successfully transformed into C. trachomatis. Using Northern blot analysis, we confirmed that these tagged RNA constructs were detected and that the sRNA levels at 1 h after aTc induction were comparable to sRNA levels at 2 or 6 h after aTc induction (Fig. S5B).

10.1128/mbio.00864-22.5 FIG S5 Aptamer affinity purification of MS2-CtrR3. (A) The secondary structures of MS2-Control (MS2-RnpB T1) and MS2-CtrR3 were predicted bioinformatically using the RNAfold software. The free energy of the thermodynamic ensemble is shown (ΔG). (B) Northern blot of lysates from MS2-Control and MS2-CtrR3 infected HeLas that were induced 1, 2, or 6 h prior to collection at 30 hpi. Blot was probed with oligonucleotide against the MS2 sequence; 5S rRNA is shown as loading control. (C) Northern blot analysis of the whole-cell lysates (WCL) and eluted materials (Elu) of MS2-Control and MS2-CtrR3 infected HeLas that were induced at 29 hpi with aTc and collected at 30 hpi for MS2-affinity purification. Northern blot was probed with oligonucleotide against the MS2 sequence. Download FIG S5, PDF file, 1.1 MB. Copyright © 2022 Wang et al. 2022 Wang et al. https://creativecommons.org/licenses/by/4.0/ This content is distributed under the terms of the Creative Commons Attribution 4.0 International license.

**FIG 6:**
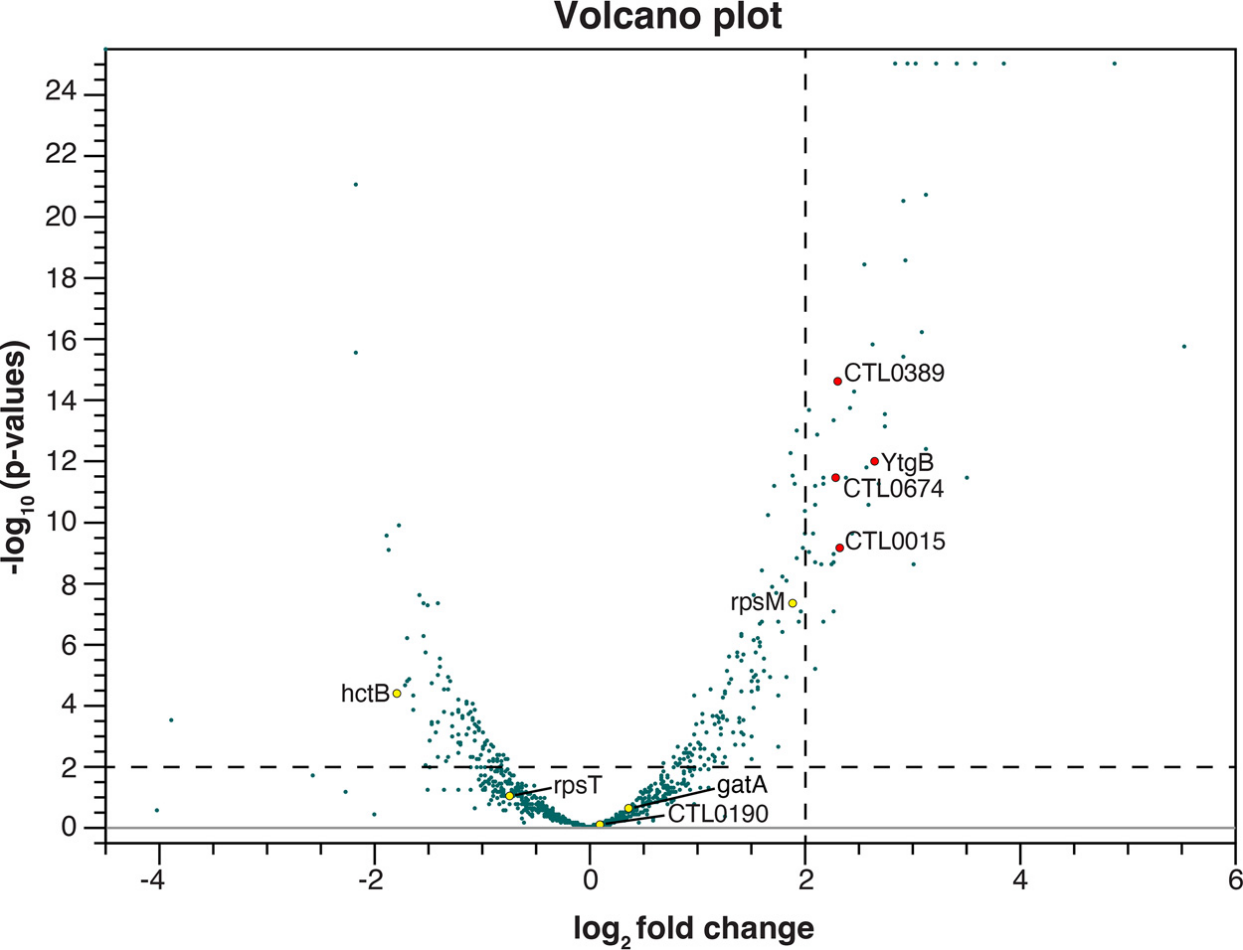
MAPS identifies putative mRNA targets of CtrR3. Volcano plot demonstrating differential enrichment of the MS2-Control (left) versus MS2-CtrR3 (right) (*n* = 2). Cutoff for enrichment was set at Log_2-fold_ ≥ 2 and *P*-value was set at ≤ 0.01 as indicated by the dashed lines. Enriched transcripts are marked with red dots. Also marked are unenriched transcripts that had been bioinformatically predicted to interact with CtrR3 target recognition sequence at the RBS (yellow dots).

We then performed MS2-affinity purification coupled with RNA sequencing (MAPS) on these transformants (20–22). We infected HeLa cells with MS2-CtrR3 or MS2-Control transformants and induced MS2-sRNAs expression with aTc for 1 h before lysing the cells at 30 hpi. We captured MS2-sRNAs and their interacting mRNAs using MS2-Maltose binding fusion proteins (MS2-MBP) that were bound to amylose resins. We verified that MS2-CtrR3 and MS2-Control were enriched in the eluted samples compared to the respective whole-cell lysate (Fig. S5C), and analyzed the eluates by RNA-seq. We then performed a differential expression analysis to identify RNAs that were enriched in the MS2-CtrR3 library compared to the MS2-Control library. Using an enrichment cutoff log_2-fold_ change ≥ 2 (*P*-value ≤ 0.01), we identified 52 transcripts that were enriched in the MS2-CtrR3 library (Fig. 6).

**(ii) Bioinformatic prioritization scheme.** We performed a bioinformatics analysis to prioritize these CtrR3-interacting RNAs and to select likely mRNA targets of CtrR3. Using the program IntaRNA (23–26), which predicts sRNA-mRNA target base-pairing, we found that 34 of the 52 mRNA hits showed sequence complementarity to the CtrR3 seed region (Table S1). We focused on four of these mRNAs, YtgB, CTL0389, CTL0015 and CTL0674, because they contained predicted CtrR3-interacting sequences located at their RBS, which is the site where sRNAs frequently bind to regulate protein expression (Fig. 6, Table 1).

**TABLE 1:** List of candidate mRNA targets of CtrR3 tested in this study

Gene name	Predicted mRNA binding sites (5′ to 3′)
*ctl0389* ^*a*^	(−16)C**GGGAGGA**GAG(−6)
*ytgB* ^*a*^	(−17)A**UUGGGAGG**GA(−7)
*ctl0015* ^*a*^	(−12)A**GGGAGG**GGAU(−2)
*ctl0674* ^*a*^	(−9)U**UUUGGGAG**A(+1)
*gatA^*b*^*	(−51)G**UUGGGAGGA**UU(−40)
*rpsT* ^*b*^	(−13)A**UUGGGAG**AGAUU(−1)
*rpsM* ^*b*^	(−15)A**GGGAGG**C(−8)
*ctl0190* ^*b*^	(−10)G**GGGAGGA**GGA(+1)
*hctB* ^*b*^	(+9)G**UUGGGAG**U **AC** A **A** (+21)

a Enriched in MAPS.

b Not enriched in MAPS.

10.1128/mbio.00864-22.8 TABLE S1 List of transcripts enriched in MAPS. Download Table S1, XLSX file, 0.02 MB. Copyright © 2022 Wang et al. 2022 Wang et al. https://creativecommons.org/licenses/by/4.0/ This content is distributed under the terms of the Creative Commons Attribution 4.0 International license.

**(iii) Functional testing with translational fusion reporter assays.** We tested these four candidates in translational fusion reporter assays, first in E. coli (27), then in Chlamydia. For the E. coli analysis, we included five negative controls, which were C. trachomatis mRNAs predicted to have sequence complementarity to the CtrR3 seed region at or near the RBS, by the sRNA target prediction program TargetRNA2 (28), but not recovered in our MAPS analysis (Fig. 6, Table 1). For each of these nine mRNA candidates, we constructed a translational fusion reporter containing the region from −50 to +30, relative to the start codon, fused upstream of *gfp*. Co-expression of these translational fusion reporters with CtrR3 in E. coli decreased GFP levels for YtgB and CTL0389, but not for CTL0015, CTL0674 or the five control mRNAs (Fig. S6).

10.1128/mbio.00864-22.6 FIG S6 E. coli translational fusion assays identify YtgB and CTL0389 as potential targets of CtrR3. (A) Western blots of lysates from E. coli co-expressing CtrR3 together with the reporter construct in which GFP was fused to of each candidate mRNA targets. CtrR3 was first expressed for 30 minutes with 200 ng/mL aTc. Subsequently, 0.02% arabinose was used to induce expression of GFP reporter constructs. The level of GFP is shown. E. coli GroEL served as a loading control. (B) Quantification of the western blots in (A). GFP was normalized first to GroEL and then to the respective uninduced controls. Data are mean ± SEM (*n* = 3); ***, *P ≤ *0.001. (C) Western blot analysis of lysates from E. coli co-expressing CtrR3 together with 6 putative mRNA target sequences fused to GFP. Expression of CtrR3 or the translational fusion proteins was induced with aTc or arabinose, respectively. GFP levels are shown. GroEL served as a loading control. Download FIG S6, PDF file, 0.5 MB. Copyright © 2022 Wang et al. 2022 Wang et al. https://creativecommons.org/licenses/by/4.0/ This content is distributed under the terms of the Creative Commons Attribution 4.0 International license.

**FIG 7:**
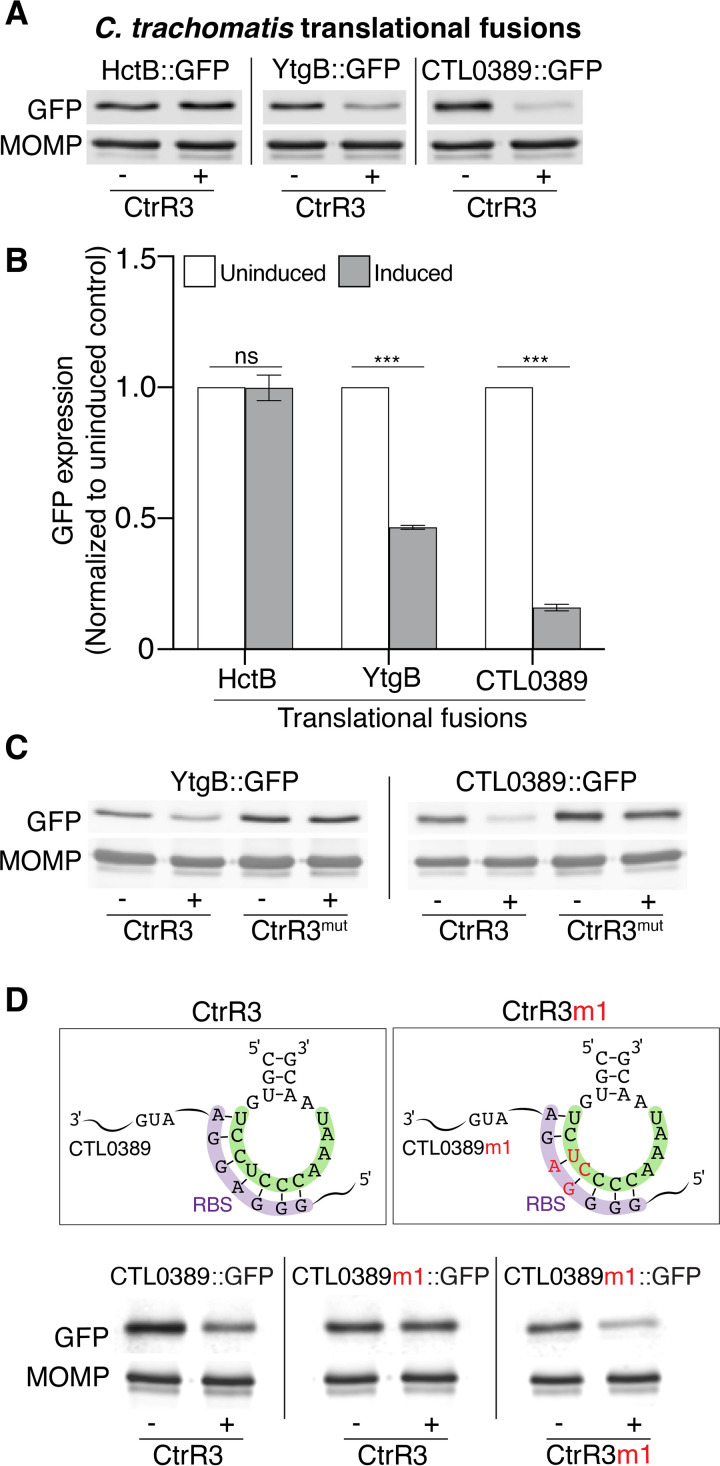
C. trachomatis translational fusion assays validate YtgB and CTL0389 as CtrR3 targets. (A) Representative Western blots of lysates from HeLa cells infected with transformants that co-expressed CtrR3 together with HctB, YtgB, or CTL0389 translational fusion proteins. To minimize deleterious effects of CtrR3 overexpression, samples were induced with aTc at 20 hpi and analyzed at 24 hpi. The level of GFP is shown. MOMP served as a loading control for Chlamydia. (B) Quantification of the Western blots in (A). GFP was normalized first to MOMP and then to the respective uninduced controls. Data are mean ± SEM (*n* = 3); ***, *P ≤ *0.001. (C) Western blots of lysates from HeLa cells infected with transformants that co-expressed the translational fusion proteins with either CtrR3 or CtrR3^mut^. sRNA overexpression was induced as described in (A). The level of GFP is shown, and MOMP served as a loading control. (D) Top: diagram of the compensatory mutations that were made to CtrR3 and the CTL0389 translational fusion. Each box shows the seed region (highlighted in green) of CtrR3 or CtrR3m1 interacting with the RBS (highlighted in purple) of either wild type CTL0389 or CTL0389m1 translational fusion mRNAs. Bottom: representative Western blots of lysates from HeLa cells infected with three different transformants that co-expressed CtrR3 together with either the CTL0389 or CTL0389m1 translational fusions, or CtrR3m1 together with the CTL0389m1 translational fusion. sRNA overexpression was induced as described in (A). The level of GFP is shown, and MOMP served as a loading control.

To confirm that YtgB and CTL0389 are bona fide CtrR3 targets, we developed an analogous translational fusion assay in C. trachomatis (Fig. S7A). This analysis required generation of a C. trachomatis transformant for each sRNA-target pair that we tested. As proof of principle, we first showed that overexpression of IhtA downregulated GFP reporter expression for its known target HctA, but not for the negative-control HctB (Fig. S7B, C). We then tested CtrR3 overexpression and found that it significantly decreased GFP reporter expression for YtgB and CTL0389 (Fig. 7A and B). For both these targets, the level of downregulation was proportional to the aTc concentration used for induction (data not shown). Importantly, GFP expression was not decreased when either YtgB and CTL0389 were co-expressed with CtrR3^mut^, which contains a disrupted target recognition sequence (Fig. 7C). These data confirm our E. coli translational fusion results and provide strong evidence that YtgB and CTL0389 are mRNA targets of CtrR3 in C. trachomatis.

10.1128/mbio.00864-22.7 FIG S7 Development of a Chlamydia translational fusion assay. (A) Plasmid map of pBOMB5-tet-sRNA translational fusion. The −50 to +30 region, relative to the translational start site, of the mRNA targets were fused upstream of the *gfp* in the pBOMB5 plasmid. Translational fusion is under the control of the Neisseria meningitidis constitutive promoter. (B) Representative western blots of lysates from HeLa cells infected with Chlamydia transformants that co-expressed IhtA together with either HctA or HctB translational fusion proteins. Infected cells were induced with aTc at 20 hpi and lysates were collected at 24 hpi to avoid the deleterious effects of IhtA overexpression. The level of GFP is shown. MOMP served as a loading control for Chlamydia. (C) Quantification of the western blot in (A). GFP was normalized first to MOMP, then to the respective uninduced controls. Data are mean ± SEM (*n* = 3); *, *P ≤ *0.05. Download FIG S7, PDF file, 0.5 MB. Copyright © 2022 Wang et al. 2022 Wang et al. https://creativecommons.org/licenses/by/4.0/ This content is distributed under the terms of the Creative Commons Attribution 4.0 International license.

We also performed reciprocal mutation experiments to test if CtrR3 directly interacts with CTL0389. In initial experiments, we made mutations in the RBS of the CTL0389 translational fusion reporter to complement the CtrR3^mut^ seed region (5′-GGGAGG-3′ to 5′-GAAAGG-3′). However, CtrR3^mut^ did not downregulate this version of CTL0389 reporter (data not shown). We then generated a new reciprocal pair by making two CT→TC mutations in the seed region of CtrR3 (CtrR3m1) and introducing reciprocal mutations in the RBS of the CTL0389 GFP reporter (CTL0389m1::GFP) (Fig. 7D). Overexpression of CtrR3m1, but not wild type CtrR3, decreased CTL0389m1 GFP reporter expression, providing genetic evidence of base-pairing between CtrR3 and CTL0389 mRNA.

## DISCUSSION

In this study, we developed a novel sRNA overexpression system in Chlamydia to investigate the function of chlamydial sRNAs in their native environment. We used this genetic system to screen 2 known and 11 uncharacterized chlamydial sRNAs for deleterious effects on the intracellular infection. We then combined our genetic approach with biochemical, bioinformatic, mutational and functional analyses to identify the target recognition sequence and mRNA targets of CtrR3, a previously uncharacterized sRNA.

Until now, functional studies of chlamydial sRNAs have been performed in E. coli but not in Chlamydia itself. The main impediment has been that Chlamydia genetic studies have not been possible until recently, and new methodologic innovations, such as transformation of Chlamydia, remain inefficient (29, 30). Studies in E. coli have demonstrated that two C. trachomatis sRNAs, IhtA and CTIG270, regulate their respective mRNA targets HctA and FtsI (Pbp3). While useful, this heterologous approach has two limitations: (i) target recognition between a chlamydial sRNA and its target transcripts may be different because Chlamydia lacks the RNA chaperone Hfq, which stabilizes sRNA-mRNA target interactions in E. coli (31); and (ii) potential roles of a chlamydial sRNA in the unique chlamydial developmental cycle cannot be investigated.

Our inducible sRNA overexpression system has a number of advantages. Most importantly, it allows a sRNA to be studied in the context of the chlamydial sRNA machinery and in the native environment of an infected host cell. Its inducible design allows the level and timing of sRNA expression to be controlled by adjusting the concentration and duration of aTc induction. Furthermore, it is versatile and can be applied to investigate sRNA target recognition and sRNA-mRNA interactions with functional studies. One experimental consideration is that this approach requires familiarity and expertise with Chlamydia genetics, which remains work-intensive. A valuable complementary genetic strategy would be to knock out the endogenous sRNA gene. However, targeted gene deletion is not suitable for studying essential genes, which is an issue for Chlamydia since this obligate intracellular bacterium has a greatly reduced genome that contains a large proportion of essential genes (32, 33). An alternative approach would be to knockdown the sRNA with the inducible CRISPRi system that has recently been developed and optimized (32, 34). This approach blocks transcription by targeting the promoter, and thus may have limitations if the sRNA is processed from an mRNA or pre-tRNA transcript, as may be the case for IhtA and CtrR3 (14, 15).

Our sRNA genetic system allowed us to identify chlamydial sRNAs whose overexpression had deleterious effects on the intracellular infection. We used a progeny defect as our read-out, but the same sRNA overexpression transformants can also be screened for other phenotypes, such as alteration of a known Chlamydia-host interaction. In our study, four sRNAs produced a progeny defect when overexpressed, suggesting that they each have an important role in the developmental cycle. This conclusion is supported by published reports showing that two of these four sRNAs, IhtA and CTIG270, regulate proteins with important functions in the Chlamydia infection. In studies with E. coli, IhtA decreased levels of HctA, which controls RB-to-EB conversion, and CTIG270 reduced the expression of FtsI, which is involved in chlamydial division (14, 16, 17). CtrR3 and CtrR7 are the two other sRNAs with a strong overexpression phenotype in our screen. They are uncharacterized *trans*-encoded sRNAs that caused a much greater progeny defect than IhtA or CTIG270. We did not detect progeny defects with nine of the sRNAs that we tested, but we cannot exclude their potential involvement in Chlamydia-host interactions or pathogenesis *in vivo*. It is also possible that this lack of a progeny defect is due to inefficient sRNA expression for seven out of the nine sRNAs.

Our analysis of CtrR3 and CtrR7 shows that overexpression of these two sRNAs affected different steps in the C. trachomatis developmental cycle. Based on assays of chlamydial genome number, progeny production, late gene expression and ultrastructural analysis, we propose that overexpression of CtrR3 disrupted RB-to-EB conversion because its effect on chlamydial genome number was small compared to the large reduction in infectious progeny. In contrast, overexpression CtrR7 mainly blocked RB replication, with the reductions in genome copies and progeny being of similar magnitude. These differential effects of CtrR3 and CtrR7 overexpression demonstrate that (i) these sRNAs most likely regulate different mRNA targets; (ii) the overexpression phenotypes are specific for each sRNA and not due to general toxicity; and (iii) it is possible to narrow down the cause of the progeny defect to a specific step in the developmental cycle. Our analysis does not, however, reveal how CtrR3 and CtrR7 overexpression, respectively, inhibit RB-to-EB conversion and RB replication, which they could do through direct or indirect mechanisms. Further studies are ongoing to delineate the roles of CtrR3 and CtrR7 in the C. trachomatis developmental cycle.

We utilized our sRNA genetic system in multiple complementary approaches to identify the mRNA targets of a chlamydial sRNA. For example, it allowed us to overexpress an MS2-tagged CtrR3 so that we could use MAPS as an unbiased method to identify interacting mRNA targets. This approach is attractive for chlamydial sRNA target identification because it does not rely on a sRNA chaperone protein, such as Hfq, as the bait to capture a sRNA and its bound targets. Our genetic approach also allowed us to identify the target recognition sequence of CtrR3, which we then used to prioritize candidate mRNA targets based on sequence complementary to the seed region. In this analysis, we focused on complementarity to the RBS (11, 12), but there is also precedent for a sRNA to bind to the coding region of its mRNA targets (22, 35, 36). Our MAPS analysis also recovered mRNAs lacking complementarity to the CtrR3 seed region, which are less likely to be bona fide targets. Since interactions identified through MAPS may be direct or indirect (22, 37), it is important to confirm the regulation of the sRNA on a candidate mRNA target.

We paired our sRNA overexpression approach with a translational fusion reporter to test and validate candidate mRNA targets. This reporter approach is commonly used in other bacteria to demonstrate that a sRNA regulates specific mRNA targets (38) and can distinguish between direct and indirect targets identified by MAPS (37). However, translational fusion assays in Chlamydia are labor-intensive because a transformant with a customized translational reporter has to be generated for each candidate target. For this reason, we first tested candidate targets with a translational fusion reporter in E. coli and then used the C. trachomatis translational fusion for confirmation. Using this approach, we identified YtgB, an ATPase for iron transport, and CTL0389, an uncharacterized inclusion membrane protein, as likely mRNA targets of CtrR3 (39, 40). We did not pursue CTL0015 and CTL0674, because they were negative in the E. coli translational fusion assay, but it remains possible that they could be targets of CtrR3 in C. trachomatis since the two bacteria have differences in their sRNA machinery. We do not yet know if CtrR3 has other mRNA targets and which target(s) may mediate its observed progeny defect.

Our studies of CtrR3 target recognition shed light on how C. trachomatis sRNAs interact with their mRNA targets in the absence of Hfq. Small RNAs in Mycobacterium, and other Gram-positive bacteria that lack Hfq and another RNA chaperone ProQ, are proposed to use C-rich motifs to interact with their mRNA targets (41). For example, Mycobacterium sRNA 6C, which is named after the 6 C-nucleotides in its recognition motif, requires 5 of these 6 C-nucleotides to regulate its target (42). Our translational fusion studies with reciprocal compensatory mutations showed that an intact 5 C/G base-pairing was necessary for functional interactions between CtrR3 and the CTL0389 translational fusion reporter (Fig. 7D). These findings are consistent with the E. coli studies conducted by Grieshaber et al., who showed that interactions between IhtA and HctA mRNA required 5 G/C interactions. This observation led these authors to propose that perfect complementarity with G/C-rich base-pairing is important for Chlamydia sRNA-mRNA binding to compensate for the absence of Hfq (43).

In summary, we have developed a Chlamydia sRNA overexpression system that provides a genetic means to study the function of a chlamydial sRNA during the intracellular infection. This approach is particularly suited for investigating the roles of sRNAs in the control of Chlamydia-specific functions, such as the developmental cycle, that cannot be studied in a heterologous system. Our overexpression system is powerful because it can be (i) readily used to screen sRNAs for effects on the infection; (ii) combined with mutational analyses to identify and validate the sRNA target seed region; and (iii) used together with an unbiased approach, such as MAPS, followed by bioinformatic prediction and functional testing to identify candidate mRNA targets. In principle, a chlamydial sRNA could even be modified to recognize a nonnative mRNA target, as the basis for a novel conditional protein knockdown method in Chlamydia, as has been done in E. coli (44, 45). Thus, our sRNA overexpression system represents an advance for studying an important posttranscriptional mechanism of gene regulation and will help to elucidate the molecular mechanisms that control the intracellular Chlamydia infection.

## MATERIALS AND METHODS

### DNA oligonucleotides and plasmids.

DNA oligonucleotides and plasmids can be found in the Supplemental Materials, Table S2. Details on plasmids construction is described in the Supplemental Materials, Text S1.

10.1128/mbio.00864-22.9 TABLE S2 DNA oligonucleotides and plasmids used in this study. Download Table S2, XLSX file, 0.02 MB. Copyright © 2022 Wang et al. 2022 Wang et al. https://creativecommons.org/licenses/by/4.0/ This content is distributed under the terms of the Creative Commons Attribution 4.0 International license.

10.1128/mbio.00864-22.1 TEXT S1 Supplemental Materials and Methods. Download TEXT S1, DOCX file, 0.03 MB. Copyright © 2022 Wang et al. 2022 Wang et al. https://creativecommons.org/licenses/by/4.0/ This content is distributed under the terms of the Creative Commons Attribution 4.0 International license.

### Antibodies used in this study.

Primary antibodies used were polyclonal rabbit anti-HctA and polyclonal rabbit anti-HctB (gifts from Ted Hackstadt), monoclonal mouse anti-MOMP (gift from Ellena Peterson), polyclonal rabbit anti-OmcB (gift from Guangming Zhong), monoclonal mouse anti-GFP (11814460001, Roche), polyclonal rabbit anti-GroEL (G6532, Sigma-Aldrich), and mouse anti-GAPDH (sc-47724, Santa Cruz). Secondary antibodies were goat anti-rabbit IgG LI-COR IRDYE 680 (926-680-71, Fisher Scientific) and goat anti-mouse IgG LI-COR IRDye 800 (926-32210; Fisher Scientific). Membranes were imaged on Odyssey CLx LI-COR machine.

### Cell culture and Chlamydia infection.

HeLa cells were obtained from ATCC and cultured at 37°C and 5.0% CO_2_ in DMEM (11995-065, Gibco) supplemented with 10% FBS (S1155, Atlanta Biologicals). McCoy cells (ATCC) were also cultured in a similar condition.

Chlamydia infections were done by infecting near-confluent cell monolayers with C. trachomatis serovar L2 (ATCC) or Chlamydia transformants at an MOI of 3 in SPG (200 mM sucrose, 20 mM sodium phosphate and 5 mM glutamate; pH 7.2) followed by centrifugation at 700 × *g* for 1 h at room temperature. After centrifugation, the inoculum was removed and replaced with DMEM containing 10% FBS. For induction of mCherry or sRNAs expression, infected cells were incubated with 50 ng/mL of aTc (94664, Supelco) in complete DMEM media at the indicated times. For experiments with aTc inductions starting at 1 hpi, aTc was replenished at 16 hpi due to the short half-life of aTc at 37°C (46).

### Chlamydia transformation.

Transformation of C. trachomatis serovar L2 was performed as previously described (47). In brief, Chlamydia EBs were incubated with 10 μg of plasmid and CaCl_2_ buffer (10 mM TRIS pH 7.4 in 50 mM CaCl_2_), followed by spin infection of a cell monolayer. The inoculum was then removed and replaced with DMEM containing 10% FBS. At 10 hpi, the medium was replaced with complete DMEM containing 10 μg/mL of ampicillin (A9518, Sigma-Aldrich). At around 48 hpi, the infected host cell monolayer was disrupted via glass beads, with the collected EBs being used to infect a new cell monolayer. After this second spin infection, the infected cells were immediately incubated in complete DMEM containing 10 μg/mL of ampicillin and 1 μg/mL of cycloheximide (NC9651091, Chem Service Inc.). This infection was labeled as passage 1 (P1). The previous two steps were repeated until P3, resulting in a selected population of Chlamydia transformants. To obtain a clonal population of transformants, EBs from P3 underwent two rounds of plaque purifications in McCoy cells as previously described (48). For the studies reported here, we generated a total of 27 C. trachomatis transformants.

### E. coli culture conditions and co-expression study.

E. coli strain DHFα (NEB5-alpha) were grown in LB (Miller) under aerobic conditions at 37°C. Where appropriate, antibiotics were used at the following concentrations: 100 μg/mL ampicillin (A9518, Sigma-Aldrich) and 25 μg/mL chloramphenicol (C0378, Sigma-Aldrich). Co-expression studies were conducted by co-transforming E. coli with the high-copy pRSETC plasmid containing the sRNA under the Tet-inducible promoter and the low-copy pBAD33.1 plasmid containing the translational fusion protein under the control of an arabinose-inducible promoter. Overnight culture of the co-transformed E. coli was diluted to an OD_600_ of 0.1 and grown to an OD_600_ of 0.5. sRNA expression was induced with 200 ng/mL aTc for 30 min, followed by induction of the translational fusion protein expression with 0.02% arabinose. Bacteria were collected 90 min post arabinose induction. Lysates, prepared through incubation 2% SDS, were subjected to SDS-PAGE and Western blot analysis.

### Progeny assay.

Progeny assays were performed as previously described (49). In brief, at the indicated times, Chlamydia*-*infected cells were washed with 1×PBS and collected in SPG to harvest infectious EBs from the primary infection. Samples were subjected to one cycle of freeze-thaw to lyse the host cells, then serially diluted in SPG and used to reinfect a new monolayer of HeLa cells in the absence of aTc. At 27 hpi, cells were fixed with ice-cold methanol, followed by visualization of chlamydial inclusions with mouse anti-MOMP antibodies (gift from Ellena Peterson, UC Irvine) using immunofluorescence microscopy. The number of inclusions, determined in 10 fields of view using a 20× objective, was used to calculate the total number of infectious progeny (IFU/mL). Progeny per cell was determined by dividing IFU/mL by the number of host cells present at the time of the infection, which was determined through counting trypsinized cells on a hemocytometer.

### Statistical analyses.

For each experiment, 3 independent biological replicates were performed, and the results are presented as mean ± SEM. Data were analyzed by unpaired, two-tailed *t* tests with Welch’s correction on Graph Pad PRISM software version 8.

## References

[1] CDC. 2021. STI prevalence, incidence, and cost estimates infographic. Centers for Disease Control and Prevention https://www.cdc.gov/std/statistics/prevalence-2020-at-a-glance.htm.

[2] Annual statistics from the National Notifiable Diseases Surveillance System (NNDSS). https://wonder.cdc.gov/nndss/nndss_annual_tables_menu.asp.

[3] Batteiger BE, Tan M. 2019. Chlamydia trachomatis (trachoma and urogenital infections), p 2301–2319. *In* Bennett JE, Dolin R, Blaser MJ (ed), Mandell, Douglas, and Bennett’s principles and practice of infectious diseases, 9th ed Elsevier Inc., Philadelphia, PA.

[4] Moulder JW. 1991. Interaction of chlamydiae and host cells in vitro. Microbiol Rev 55:143–190. doi:10.1128/mr.55.1.143-190.1991. 2030670 PMC372804

[5] Lee JK, Enciso GA, Boassa D, Chander CN, Lou TH, Pairawan SS, Guo MC, Wan FYM, Ellisman MH, Sütterlin C, Tan M. 2018. Replication-dependent size reduction precedes differentiation in Chlamydia trachomatis. Nat Commun 9:45. doi:10.1038/s41467-017-02432-0. 29298975 PMC5752669

[6] Belland RJ, Zhong G, Crane DD, Hogan D, Sturdevant D, Sharma J, Beatty WL, Caldwell HD. 2003. Genomic transcriptional profiling of the developmental cycle of Chlamydia trachomatis. Proc Natl Acad Sci USA 100:8478–8483. doi:10.1073/pnas.1331135100. 12815105 PMC166254

[7] Rosario CJ, M T. 2020. Chapter 10: chlamydia gene regulation, 219–240. *In* Chlamydia Biology: from genome to disease. Caister Academic Press, Norfolk, UK.

[8] Brickman TJ, Barry CE, Hackstadt T. 1993. Molecular cloning and expression of hctB encoding a strain-variant chlamydial histone-like protein with DNA-binding activity. J Bacteriol 175:4274–4281. doi:10.1128/jb.175.14.4274-4281.1993. 7687246 PMC204866

[9] Hackstadt T, Baehr W, Ying Y. 1991. Chlamydia trachomatis developmentally regulated protein is homologous to eukaryotic histone H1. Proc Natl Acad Sci USA 88:3937–3941. doi:10.1073/pnas.88.9.3937. 2023942 PMC51568

[10] Gottesman S, Storz G. 2011. Bacterial small RNA regulators: versatile roles and rapidly evolving variations. Cold Spring Harb Perspect Biol 3:a003798–a003798. doi:10.1101/cshperspect.a003798. 20980440 PMC3225950

[11] Storz G, Vogel J, Wassarman KM. 2011. Regulation by Small RNAs in Bacteria: expanding Frontiers. Mol Cell 43:880–891. doi:10.1016/j.molcel.2011.08.022. 21925377 PMC3176440

[12] Waters LS, Storz G. 2009. Regulatory RNAs in bacteria. Cell 136:615–628. doi:10.1016/j.cell.2009.01.043. 19239884 PMC3132550

[13] Georg J, Hess WR. 2011. cis-antisense RNA, another level of gene regulation in bacteria. Microbiol Mol Biol Rev 75:286–300. doi:10.1128/MMBR.00032-10. 21646430 PMC3122628

[14] AbdelRahman YM, Rose LA, Belland RJ. 2011. Developmental expression of non-coding RNAs in Chlamydia trachomatis during normal and persistent growth. Nucleic Acids Res 39:1843–1854. doi:10.1093/nar/gkq1065. 21051342 PMC3061062

[15] Albrecht M, Sharma CM, Reinhardt R, Vogel J, Rudel T. 2010. Deep sequencing-based discovery of the Chlamydia trachomatis transcriptome. Nucleic Acids Res 38:868–877. doi:10.1093/nar/gkp1032. 19923228 PMC2817459

[16] Grieshaber NA, Grieshaber SS, Fischer ER, Hackstadt T. 2006. A small RNA inhibits translation of the histone-like protein Hc1 in Chlamydia trachomatis. Mol Microbiol 59:541–550. doi:10.1111/j.1365-2958.2005.04949.x. 16390448

[17] Ouellette SP, Karimova G, Subtil A, Ladant D. 2012. Chlamydia co-opts the rod shape-determining proteins MreB and Pbp2 for cell division. Mol Microbiol 85:164–178. doi:10.1111/j.1365-2958.2012.08100.x. 22624979

[18] Bauler LD, Hackstadt T. 2014. Expression and targeting of secreted proteins from Chlamydia trachomatis. J Bacteriol 196:1325–1334. doi:10.1128/JB.01290-13. 24443531 PMC3993338

[19] Lorenz R, Bernhart SH, Höner Zu Siederdissen C, Tafer H, Flamm C, Stadler PF, Hofacker IL. 2011. ViennaRNA Package 2.0. Algorithms Mol Biol 6:26. doi:10.1186/1748-7188-6-26. 22115189 PMC3319429

[20] Mercier N, Prévost K, Massé E, Romby P, Caldelari I, Lalaouna D. 2021. MS2-affinity purification coupled with RNA sequencing in Gram-positive bacteria. JoVE doi:10.3791/61731. 33720114

[21] Lalaouna D, Desgranges E, Caldelari I, Marzi S. 2018. MS2-affinity purification coupled with RNA sequencing approach in the human pathogen Staphylococcus aureus. Methods Enzymol 612:393–411. doi:10.1016/bs.mie.2018.08.022. 30502950

[22] Lalaouna D, Massé E. 2015. Identification of sRNA interacting with a transcript of interest using MS2-affinity purification coupled with RNA sequencing (MAPS) technology. Genom Data 5:136–138. doi:10.1016/j.gdata.2015.05.033. 26484242 PMC4583644

[23] Mann M, Wright PR, Backofen R. 2017. IntaRNA 2.0: enhanced and customizable prediction of RNA–RNA interactions. Nucleic Acids Res 45:W435–W439. doi:10.1093/nar/gkx279. 28472523 PMC5570192

[24] Wright PR, Georg J, Mann M, Sorescu DA, Richter AS, Lott S, Kleinkauf R, Hess WR, Backofen R. 2014. CopraRNA and IntaRNA: predicting small RNA targets, networks and interaction domains. Nucleic Acids Res 42:W119–123. doi:10.1093/nar/gku359. 24838564 PMC4086077

[25] Busch A, Richter AS, Backofen R. 2008. IntaRNA: efficient prediction of bacterial sRNA targets incorporating target site accessibility and seed regions. Bioinformatics 24:2849–2856. doi:10.1093/bioinformatics/btn544. 18940824 PMC2639303

[26] Raden M, Ali SM, Alkhnbashi OS, Busch A, Costa F, Davis JA, Eggenhofer F, Gelhausen R, Georg J, Heyne S, Hiller M, Kundu K, Kleinkauf R, Lott SC, Mohamed MM, Mattheis A, Miladi M, Richter AS, Will S, Wolff J, Wright PR, Backofen R. 2018. Freiburg RNA tools: a central online resource for RNA-focused research and teaching. Nucleic Acids Res 46:W25–W29. doi:10.1093/nar/gky329. 29788132 PMC6030932

[27] Urban JH, Vogel J. 2007. Translational control and target recognition by Escherichia coli small RNAs in vivo. Nucleic Acids Res 35:1018–1037. doi:10.1093/nar/gkl1040. 17264113 PMC1807950

[28] Kery MB, Feldman M, Livny J, Tjaden B. 2014. TargetRNA2: identifying targets of small regulatory RNAs in bacteria. Nucleic Acids Res 42:W124–W129. doi:10.1093/nar/gku317. 24753424 PMC4086111

[29] Rahnama M, Fields KA. 2018. Transformation of Chlamydia: current approaches and impact on our understanding of chlamydial infection biology. Microbes Infect 20:445–450. doi:10.1016/j.micinf.2018.01.002. 29409975 PMC6070436

[30] Mueller KE, Wolf K, Fields KA. 2017. Chlamydia trachomatis transformation and allelic exchange mutagenesis. Curr Protoc Microbiol 45:11A.3.1–15. 10.1002/cpmc.31 PMC5545879 28510361

[31] De Lay N, Schu DJ, Gottesman S. 2013. Bacterial small RNA-based negative regulation: Hfq and its accomplices. J Biol Chem 288:7996–8003. doi:10.1074/jbc.R112.441386. 23362267 PMC3605619

[32] Ouellette SP. 2018. Feasibility of a conditional knockout system for Chlamydia based on CRISPR interference. Front Cell Infect Microbiol 8. doi:10.3389/fcimb.2018.00059. PMC5835046 29535977

[33] Sigalova OM, Chaplin AV, Bochkareva OO, Shelyakin PV, Filaretov VA, Akkuratov EE, Burskaia V, Gelfand MS. 2019. Chlamydia pan-genomic analysis reveals balance between host adaptation and selective pressure to genome reduction. BMC Genomics 20:710. doi:10.1186/s12864-019-6059-5. 31510914 PMC6740158

[34] Ouellette SP, Blay EA, Hatch ND, Fisher-Marvin LA. 2021. CRISPR interference to inducibly repress gene expression in Chlamydia trachomatis. Infect Immun 89. doi:10.1128/IAI.00108-21. PMC8373233 33875479

[35] Fröhlich KS, Papenfort K, Berger AA, Vogel J. 2012. A conserved RpoS-dependent small RNA controls the synthesis of major porin OmpD. Nucleic Acids Res 40:3623–3640. doi:10.1093/nar/gkr1156. 22180532 PMC3333887

[36] Carrier M-C, Lalaouna D, Massé E. 2018. Broadening the definition of bacterial small RNAs: characteristics and mechanisms of action. Annu Rev Microbiol 72:141–161. doi:10.1146/annurev-micro-090817-062607. 30200848

[37] Lalaouna D, Prévost K, Eyraud A, Massé E. 2017. Identification of unknown RNA partners using MAPS. Methods 117:28–34. doi:10.1016/j.ymeth.2016.11.011. 27876680

[38] Sharma CM, Vogel J. 2009. Experimental approaches for the discovery and characterization of regulatory small RNA. Curr Opin Microbiol 12:536–546. doi:10.1016/j.mib.2009.07.006. 19758836

[39] Thompson CC, Nicod SS, Malcolm DS, Grieshaber SS, Carabeo RA. 2012. Cleavage of a putative metal permease in Chlamydia trachomatis yields an iron-dependent transcriptional repressor. Proc Natl Acad Sci USA 109:10546–10551. doi:10.1073/pnas.1201398109. 22689982 PMC3387080

[40] Weber MM, Bauler LD, Lam J, Hackstadt T. 2015. Expression and localization of predicted inclusion membrane proteins in Chlamydia trachomatis. Infect Immun 83:4710–4718. doi:10.1128/IAI.01075-15. 26416906 PMC4645406

[41] Jørgensen MG, Pettersen JS, Kallipolitis BH. 2020. sRNA-mediated control in bacteria: an increasing diversity of regulatory mechanisms. Biochim Biophys Acta Gene Regul Mech 1863:194504. doi:10.1016/j.bbagrm.2020.194504. 32061884

[42] Mai J, Rao C, Watt J, Sun X, Lin C, Zhang L, Liu J. 2019. Mycobacterium tuberculosis 6C sRNA binds multiple mRNA targets via C-rich loops independent of RNA chaperones. Nucleic Acids Res 47:4292–4307. doi:10.1093/nar/gkz149. 30820540 PMC6486639

[43] Grieshaber NA, Tattersall JS, Liguori J, Lipat JN, Runac J, Grieshaber SS. 2015. Identification of the base-pairing requirements for repression of hctA translation by the small RNA IhtA leads to the discovery of a new mRNA target in Chlamydia trachomatis. PLoS One 10:e0116593. doi:10.1371/journal.pone.0116593. 25756658 PMC4355289

[44] Nakashima N, Tamura T, Good L. 2006. Paired termini stabilize antisense RNAs and enhance conditional gene silencing in Escherichia coli. Nucleic Acids Res 34. doi:10.1093/nar/gkl697. PMC1635301 17062631

[45] Magistro G, Magistro C, Stief CG, Schubert S. 2018. A simple and highly efficient method for gene silencing in Escherichia coli. J Microbiol Methods 154:25–32. doi:10.1016/j.mimet.2018.10.003. 30296471

[46] Politi N, Pasotti L, Zucca S, Casanova M, Micoli G, Cusella De Angelis MG, Magni P. 2014. Half-life measurements of chemical inducers for recombinant gene expression. J Biol Eng 8:5. doi:10.1186/1754-1611-8-5. 24485151 PMC3940292

[47] Wang Y, Kahane S, Cutcliffe LT, Skilton RJ, Lambden PR, Clarke IN. 2011. Development of a transformation system for Chlamydia trachomatis: restoration of glycogen biosynthesis by acquisition of a plasmid shuttle vector. PLoS Pathog 7:e1002258. doi:10.1371/journal.ppat.1002258. 21966270 PMC3178582

[48] Skipp P, Robinson J, O'Connor CD, Clarke IN. 2005. Shotgun proteomic analysis of Chlamydia trachomatis. Proteomics 5:1558–1573. doi:10.1002/pmic.200401044. 15838905

[49] Muñoz KJ, Wang K, Sheehan LM, Tan M, Sütterlin C. 2021. The small molecule H89 Inhibits Chlamydia inclusion growth and production of infectious progeny. Infect Immun 89:e00729-20. doi:10.1128/IAI.00729-20. PMC8373235 33820812

