# Supplementary figures and images for: A Reverse Genetic Approach for Studying sRNAs in Chlamydia trachomatis
Source: mBio. 2022 Jun 21;13(4):e00864-22. doi: 10.1128/mbio.00864-22 (PMC9426522; doi:10.1128/mbio.00864-22)

Figure S1

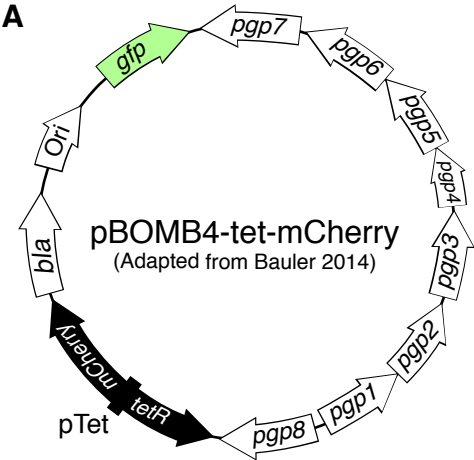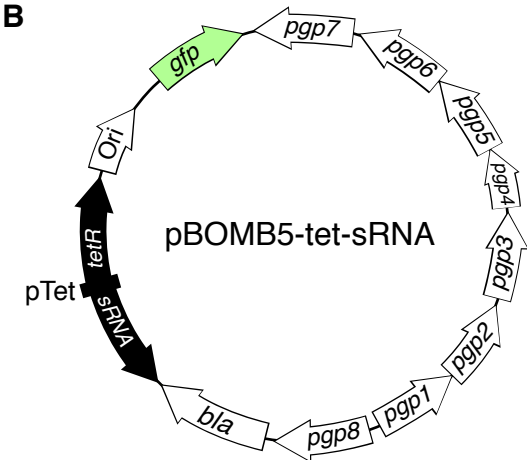

Supplement: FIG S1 [file mbio.00864-22-s0002.pdf]

Figure S2

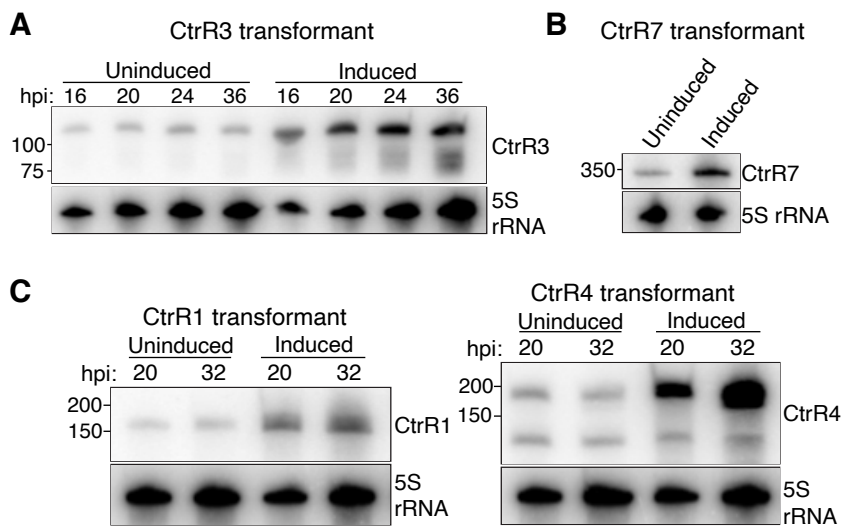

Supplement: FIG S2 [file mbio.00864-22-s0003.pdf]

Figure S3

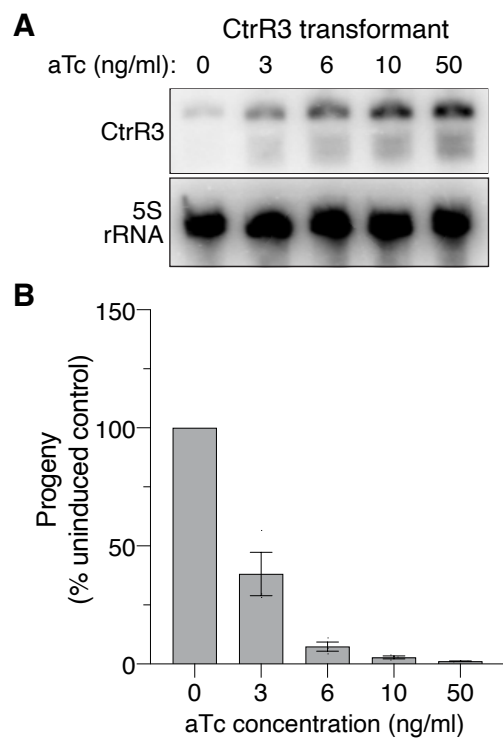

Supplement: FIG S3 [file mbio.00864-22-s0004.pdf]

Figure S4

**A**

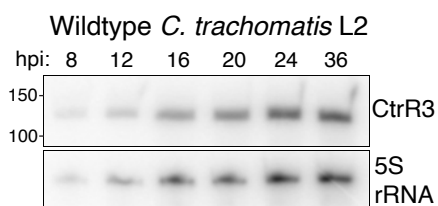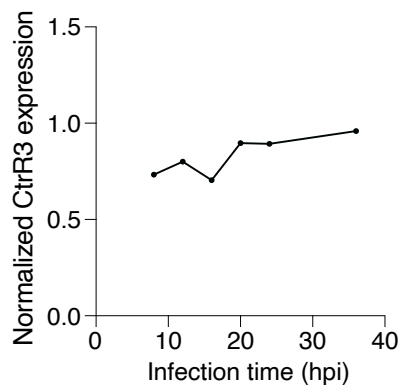

**B**

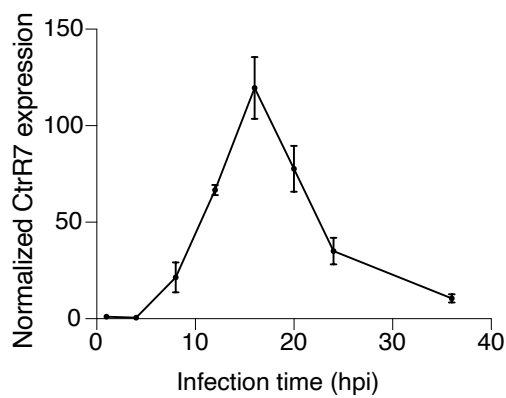

Supplement: FIG S4 [file mbio.00864-22-s0005.pdf]

Figure S5

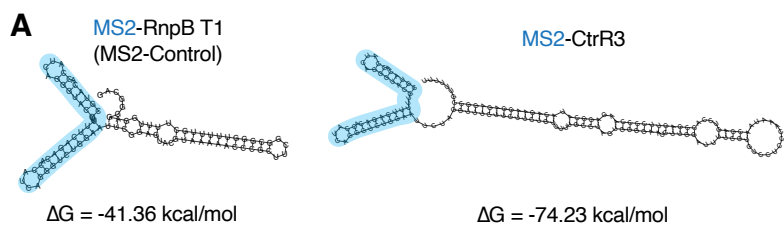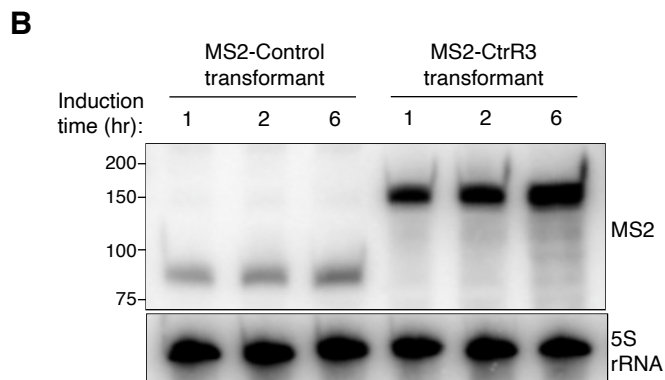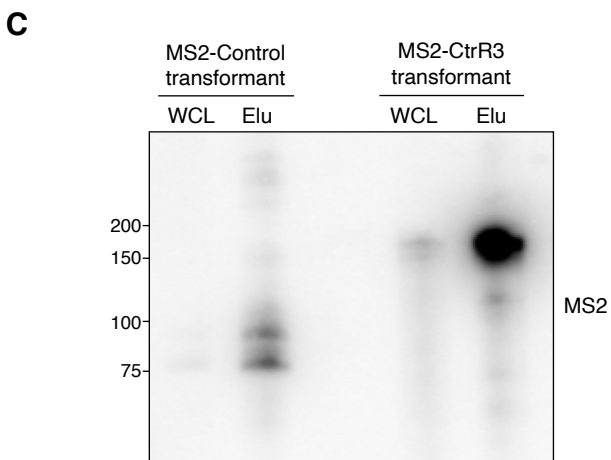

Supplement: FIG S5 [file mbio.00864-22-s0006.pdf]

Figure S6

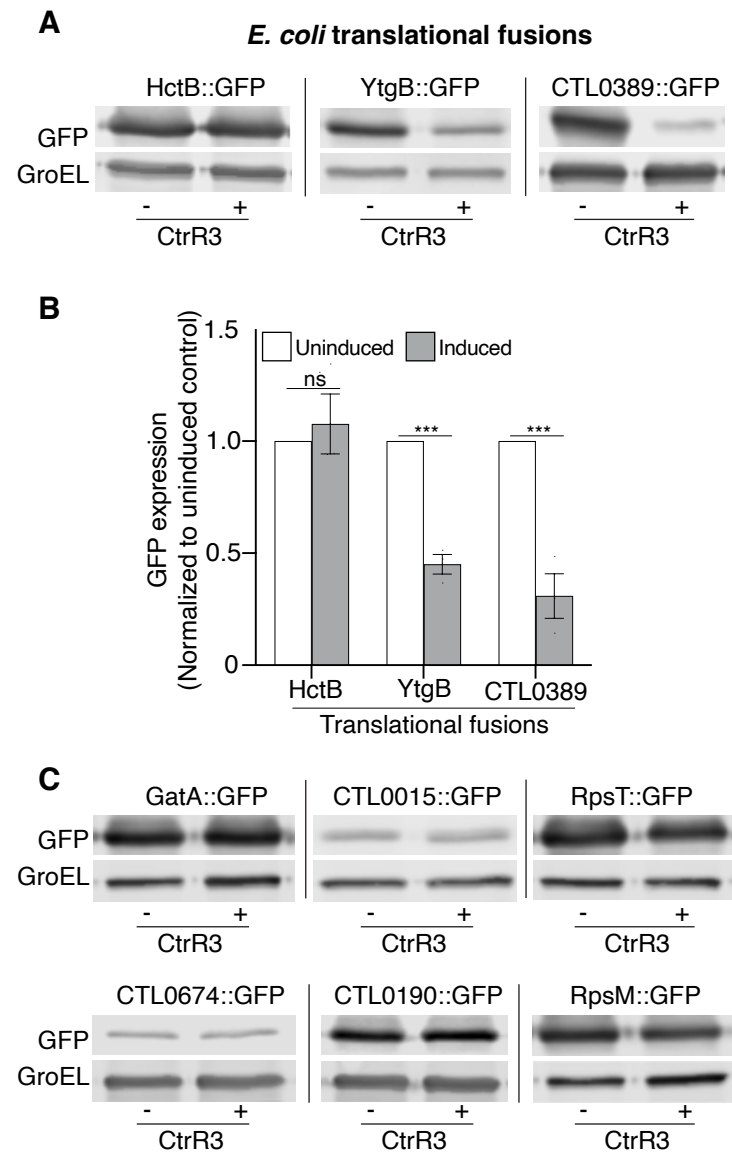

Supplement: FIG S6 [file mbio.00864-22-s0007.pdf]

Figure S7

**A**

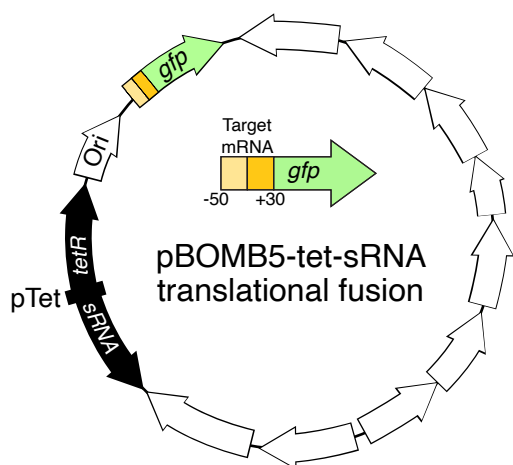

**B**

*C. trachomatis* translational fusions

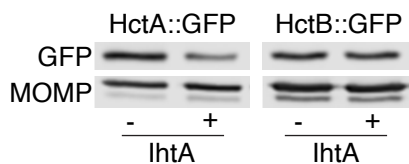

**C**

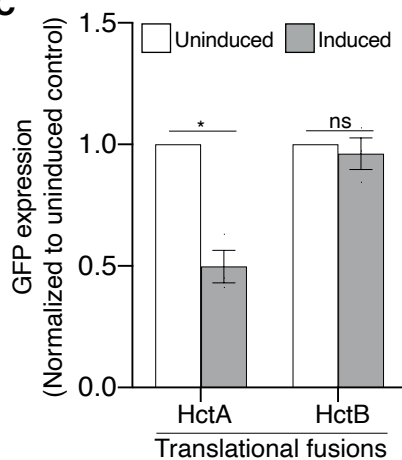

Supplement: FIG S7 [file mbio.00864-22-s0008.pdf]
