# Supplementary material for: A Reverse Genetic Approach for Studying sRNAs in Chlamydia trachomatis
Source: mBio. 2022 Jun 21;13(4):e00864-22. doi: 10.1128/mbio.00864-22 (PMC9426522; doi:10.1128/mbio.00864-22)
Supplement: TEXT S1 [file mbio.00864-22-s0001.docx]

**Supplemental materials for the manuscript “A reverse genetic approach for studying sRNAs in *Chlamydia trachomatis*”**

**Supplemental Materials and Methods**

**Plasmid construction**

All plasmids were constructed with Gibson assembly via the NEBuilder HiFi DNA assembly mastermix (E2621, NEB) and subsequently transformed into *E. coli* strain DHFα (NEB5-alpha). The plasmid constructs were then confirmed via Sanger sequencing using Retrogen Inc.

To construct pBOMB5-tet-IhtA, the Tet-inducible mCherry expression cassette was first removed from pBOMB4-tet-mCherry using primer pairs P1+P2 and P3+P4. Subsequently, IncG terminator and the IhtA overexpression cassette was placed downstream of the *bla* gene using primer pairs P1+P5, P6+P7, P8+P9, P10+P11, and P12+P4. To overexpress mCherry and other sRNAs in the pBOMB5 plasmid, primer pairs P1+P13 and P10+P4 were used to amplify the vector backbone of pBOMB5-tet-IhtA. IhtA was then replaced by using primer pairs from P14 to P41. pBOMB5-tet-MS2-CtrR3 was generated with primer pairs P1+P42 and P10+P4 using pBOMB5-tet-CtrR3 as template. The two fragments were joined by P43. pBOMB5-tet-MS2-RnpB T1 was made using primer sets P1+P44 and P45+P4 using pBOMB5-tet-MS2-CtrR3 as template. These two fragments were joined by P46. pBOMB5-tet-CtrR3^mut^ was generated with primer pairs P47+P4 and P1+P48 using pBOMB5-tet-CtrR3 as template.

pRSETC-tet-CtrR3 was constructed with primer pairs P49+P50 for the pRSETC vector backbone and P51+P52 for the CtrR3 overexpression cassette amplified from the pBOMB5-tet-CtrR3 plasmid. To generate the plasmid for arabinose-inducible translational fusion expression, pBAD33.1 vector backbone was amplified with P53+P54. GFP was amplified from the pBOMB5-tet-sRNA plasmid using primer pairs P55+P56. The -50 to +30 region of each putative mRNA targets were amplified with primer pairs P57 to P74.

The pBOMB5-tet-sRNA translational fusion plasmid was generated by amplifying the vector backbone using P1+P75 and P55+P4 using either pBOMB5-tet-IhtA or pBOMB5-tet-CtrR3 as templates. The -50 to +30 region of *hctA*, *hctB*, *ytgB*, *ctl0389* were amplified using primer pairs P76+P77, P78+P58, P79+P60, P80+P62 respectively. Plasmid with CtrR3^mut^ co-expressing with YtgB or CTL0389 translational fusion proteins were generated with primer pairs P47+P4 and P1+P48 using pBOMB5-tet-CtrR3-YtgB::GFP and pBOMB5-tet-CtrR3-CTL0389::GFP as templates, respectively. Plasmid with CtrR3 co-expressing with CTL0389m1 translational fusion protein were generated with primers pair P1+P82 and P81+P4 using pBOMB5-tet-CtrR3-CTL0389::GFP as template. The two fragments were then assembled using P83 link oligonucleotide. Plasmid with CtrR3m1 co-expressing CTL0389m1::GFP was generated with primer pairs P1+P48 and P84+P4 using pBOMB5-tet-CtrR3-CTL0389m1::GFP as template. The two fragments were assembled using P85 link oligonucleotide.

**Northern blot**

Total RNA was prepared from *Chlamydia* infected cells using TRIzol (15-596-026, Invitrogen) and chloroform with Phase Lock Gel tubes (2302830, Quantabio). Northern blot was performed as previously described^1^. Briefly, 12 µg of RNA was resolved in 10% polyacrylamide gel with Tris-Borate-EDTA (TBE) and 7M urea. Low molecular-weight ladder (N3233, NEB) was radiolabeled with (γ-^32^P)-ATP (Perkin Elmer) using polynucleotide kinase (M0201, NEB) to identify RNA sizes. RNA samples were then transferred to Hybond-N+ membrane (GERPN303N, Cytiva) and then UV cross-linked to the membrane. Membranes were prehybridized with ULTRAhyb-Oligo buffer (AM8663, Invitrogen) for 30 minutes at 42°C. The oligonucleotide probes, which can be found in Table S2A, were end labeled with γ-^32^P-ATP and incubated with the membranes overnight. The following day, membranes were washed four times with 2x SSC, 1x SSC, 0.05x SSC, and 0.025x SSC (1x SSC is 0.15M NaCl and 0.015M sodium citrate) plus 0.1% SDS. Membranes were exposed to phosphor-imager screens and visualized by Typhoon TRIO+ Imager.

**Western blot**

Cell lysates were prepared by lysing *Chlamydia-*infected cells or *E. coli* cells directly in 2% SDS, followed by boiling the samples at 95°C for 5 min as previously described^2^. Equal volumes of lysates were loaded and resolved by SDS-PAGE, followed by transferring onto nitrocellulose membranes.

**RT-qPCR and qPCR**

RT-qPCR was used to measure relative transcript levels in *Chlamydia*. Total RNA extracted from *Chlamydia*-infected cells was subjected to DNAse (FEREN0521, Thermo Scientific) treatment, followed by cDNA synthesis with qScript cDNA SuperMix (95048-025, QuantaBio). qPCR reactions were conducted on diluted cDNA with SsoAdvanced universal SYBR green supermix (1725271, Bio-Rad) and was run on a Bio-Rad thermocycler. qPCR of the *C. trachomatis 16s rRNA* gene was used as control for all samples. Primers used can be found in Table S2A. PCR efficiencies were calculated using LinRegPCR software^3^, and relative levels of mRNA abundance compared to the uninduced control samples were calculated using the Pfaffl equation^4^.

The number of chlamydial genomes per host cell was measured by qPCR. Genome copy number was determined using a standard curve generated from a plasmid containing the *C. trachomatis euo* gene^2^. The number of host cells was determined from PCR reactions of the host cell gene *gapdh*. *Chlamydia* genomes per host cell was calculated by normalizing genome copy number (*euo*) to *gapdh* values in each sample. Primer sequences to *euo* and *gapdh* can be found in Table S2A. qPCR was performed using SsoAdvanced universal SYBR green supermix and analyzed on the Bio-Rad thermocycler.

**EM**

For transmission electron microscopy analysis, *Chlamydia*-infected cells were fixed in 2% paraformaldehyde (100503-917, VWR) and 2.5% glutaraldehyde (NC9861069, Polysciences) in 0.1M cacodylate buffer for 2 hours at room temperature. Samples were processed and imaged by Dr. Wandy Beatty at Washington University, School of Medicine.

**MS2-affinity purification coupled with RNA sequencing**

MS2-affinity purification was conducted by modifying the protocol previously described^5^. Two 150 mm dishes of HeLa cells were infected with either the MS2-Control or the MS2-CtrR3 transformants at an MOI of 3. At 29 hpi, 50ng/mL of aTc was added to each plate to induce MS2-sRNA expression. At 30 hpi, infected HeLa cells were trypsinized, pelleted at 500 g for 5 minutes, and washed once in PBS. After PBS removal, the cell pellets were frozen with liquid nitrogen and stored at -80°C. Cell pellets were then thawed on ice and resuspended in Buffer A, which is composed of 20mM Tris-HCl pH 8, 150mM KCl, 1mM MgCl_2_, 60U of SUPERase (Fischer Scientific AM2694), and 1mM DTT in DEPC-treated water. Resuspended cells were transferred to Matrix B tube (MP Biomedical 116911050-CF) and lysed with the Fast Prep-24 homogenizer (MP Biomedical) at 4.0 m/s for 15 seconds. Lysates were then transferred to a new Eppendorf tube after spinning down the matrix beads at 3000 g for 5 minutes. A small fraction of the lysate (whole cell lysate, WCL) was saved for northern blot analysis.

Prior to loading the lysates onto the columns, amylose resin (NEB E8021S) was added to the Poly-prep Chromatography columns (Bio-Rad #731150). The resin was washed with Buffer A (no SUPERase added) and then incubated with 6mL of MS2-MBP (~1,200 pmol) in Buffer A. The columns were washed once with Buffer A then the lysates were loaded onto the columns. After 3 washes with Buffer A, the MS2-sRNAs were eluted with Buffer E, which is composed of 150mM KCl, 1mM DTT, and 12mM maltose in DEPC-treated water. RNA from both the WCLs and eluates were extracted with 1:1 Acid-Phenol: Chloroform: IAA (Invitrogen AM9720) followed by one chloroform wash. The RNA was then precipitated in -20°C with GlycoBlue (Thermo Scientific AM9515) and 2.5 times in volume of 100% ethanol. The next day, precipitated RNAs were pelleted and washed once with 70% ethanol. RNA pellets were dried at room temperature and resuspended in nuclease-free water. Extracted RNAs were then treated with Turbo DNase (Invitrogen AM2238) and re-extracted using the aforementioned method. From here the RNA integrity of the DNAse-treated RNAs was assessed by determining the 3':5' ratio of the cDNA^6^. After confirmation of the RNA qualities, the DNAse-treated RNAs subsequently underwent RNA sequencing (eluates only) or northern blot analysis^6^. MS2-affinity purification was done in duplicates.

MS2-enriched paired end libraries were sequenced on an Illumina NovaSeq 6000 sequencing platform in duplicate. Two MS2-CtrR3 enriched libraries were sequenced to 85 and 98 million reads each with 10 and 16 million reads, respectively, mapping to *Chlamydia trachomatis* L2 434/Bu genome (accession GCA_000068585.1) excluding reads mapped to rRNAs. Similarly, two MS2-Control libraries were sequenced to a total of 102 and 78 million reads of which 10 and 6 million reads were mapped to the *Chlamydia* genome, excluding reads mapped to rRNA. All mappings and data analysis were performed on Qiagen CLC Genomics Workbench (version 21.0.5) software with high stringency settings (mismatch cost 2, and insertion and deletion cost 3, length and similarity fraction 0.8 each, maximum number of hits for a read to 10, and minimum read count fusion table to 5). The differential expression statistical analysis was done with MS2-CtrR3 Vs. MS2-Control libraries using “Differential Expression in Two Groups” function filtering for average expression for False Discovery Rate (FDR) correction. The p-value reported are FDR p-value. Default RNA-seq analysis method in CLC Genomics Workbench software counts fragments (FPKM) instead of individual reads. This method is more accurate as only unbroken fragment pairs are assigned a read, removing low quality read fragments from the analysis.

**Bioinformatic predictions of CtrR3 targets**

IntaRNA^7–9^ was used to analyze the 52 transcripts that were enriched > 2 Log_2_-fold in MAPS analysis to identify likely mRNA targets of CtrR3. This bioinformatic tool predicts base-pairing between a query sRNA and specific candidate mRNAs entered into the program. The entire CtrR3 sequence was entered as query ncRNA in the IntaRNA prediction site. For each mRNA transcript, 50 nucleotides upstream of the start codon, the coding sequence, and 50 nucleotides downstream of the stop codon were entered into the prediction site. The mRNA sequences were obtained from *Chlamydia trachomatis* 434/Bu genome (GCA_000068585.1). The parameters were set to default except for the “Min. number of base-pairs in seed” was set to 6. The list of putative mRNA targets was first narrowed down by identifying those with sequence complementarity to the CtrR3 seed region (49-61 nt). We then prioritized mRNAs which had sequence complementarity at their RBS (i.e. A/G-rich sequences upstream of the start codon).

TargetRNA2^10^ was utilized to identify mRNA targets that were also predicted to base-pair with the CtrR3 seed region at or near the RBS but were not identified in the MAPS analysis. This bioinformatic tool predicts sites of complementarity base-pairing with the sRNA sequence in the genome, which is beneficial for when candidate mRNAs are not known. The hairpin loop sequence 5'-TGTCCTCCCAAATAAC-3' of CtrR3 was entered into the TargetRNA2 prediction site against the *Chlamydia trachomatis* 434/Bu genome (GCA_000068585.1). The constraints were set to search 50 nucleotides upstream and 30 nucleotides downstream of the translational start site. The hybridization seed was set to 6 nucleotides and P-value threshold was set to 0.5. Similar to the IntaRNA analysis, the list of predicted mRNA targets was further narrowed down by selecting for candidates that were predicted to base-pair with the seed region at the RBS, upstream of the RBS, or 5′ coding region of the mRNA.

**References**

1. Sheehan, L. M. & Caswell, C. C. A 6-Nucleotide Regulatory Motif within the AbcR Small RNAs of Brucella abortus Mediates Host-Pathogen Interactions. *mBio* **8**, e00473-17 (2017).

2. Muñoz, K. J., Wang, K., Sheehan, L. M., Tan, M. & Sütterlin, C. The Small Molecule H89 Inhibits Chlamydia Inclusion Growth and Production of Infectious Progeny. *Infection and Immunity* **89**, e00729-20 (2021).

3. Ruijter, J. M. *et al.* Amplification efficiency: linking baseline and bias in the analysis of quantitative PCR data. *Nucleic Acids Res* **37**, e45 (2009).

4. Pfaffl, M. W. A new mathematical model for relative quantification in real-time RT-PCR. *Nucleic Acids Res* **29**, e45 (2001).

5. Mercier, N. *et al.* MS2-Affinity Purification Coupled with RNA Sequencing in Gram-Positive Bacteria. *JoVE (Journal of Visualized Experiments)* e61731 (2021) doi:10.3791/61731.

6. Die, J. V., Obrero, Á., González-Verdejo, C. I. & Román, B. Characterization of the 3’:5’ ratio for reliable determination of RNA quality. *Anal Biochem* **419**, 336–338 (2011).

7. Busch, A., Richter, A. S. & Backofen, R. IntaRNA: efficient prediction of bacterial sRNA targets incorporating target site accessibility and seed regions. *Bioinformatics* **24**, 2849–2856 (2008).

8. Mann, M., Wright, P. R. & Backofen, R. IntaRNA 2.0: enhanced and customizable prediction of RNA–RNA interactions. *Nucleic Acids Research* **45**, W435–W439 (2017).

9. Wright, P. R. *et al.* CopraRNA and IntaRNA: predicting small RNA targets, networks and interaction domains. *Nucleic Acids Res* **42**, W119-123 (2014).

10. Lorenz, R. *et al.* ViennaRNA Package 2.0. *Algorithms Mol Biol* **6**, 26 (2011).
